# Phytoplankton growth and potential cyanotoxin production differ in response to nitrogen and phosphorus amendments in late summer communities from Kabetogama Lake (Minnesota, United States)

**DOI:** 10.1111/jpy.70166

**Published:** 2026-05-02

**Authors:** James H. Larson, Ryan P. Maki, Sean W. Bailey, Victoria G. Christensen, Keith A. Loftin, Erin A. Stelzer, James C. Smith, Seth A. McWhorter

**Affiliations:** ^1^ U.S. Geological Survey Upper Midwest Environmental Sciences Center La Crosse Wisconsin USA; ^2^ Voyageurs National Park International Falls Minnesota USA; ^3^ U.S. Geological Survey Upper Midwest Water Science Center St. Paul Minnesota USA; ^4^ US Geological Survey Kansas Water Science Center, Algal and Other Environmental Toxins Laboratory Lawrence Kansas USA; ^5^ U.S. Geological Survey Ohio‐Kentucky‐Indiana Water Science Center Columbus Ohio USA; ^6^ Environmental Protection Agency Region 4, Water Division Atlanta Georgia USA

**Keywords:** cyanobacteria, cyanotoxin production, Kabetogama Lake, microcystin, nitrogen, phosphorus

## Abstract

Cyanotoxins such as microcystin (MC), cylindrospermopsin, and saxitoxin are secondary metabolites that are rich in nitrogen (N). Most cyanobacteria grow best on reduced inorganic N (ammonium, NH_4_), but when NH_4_ is absent, cyanobacteria can activate physiological pathways to process other N forms (e.g., nitrate; NO_3_). Studies on some cyanobacterial cultures have indicated that expression of N stress response genes is associated with MC gene expression. If accurate, NH_4_ additions could reduce N stress and therefore cyanotoxin production. Lakes experiencing changes in N form and supply could thus experience changes in cyanobacterial toxicity independent of shifts in abundance. We performed nutrient amendments on phytoplankton communities from Kabetogama Lake (Minnesota, United States) over 2 years (four experiments total), to assess the role of N and phosphorus amendments on growth and toxicity of phytoplankton. Natural communities were collected during cyanobacterial blooms and exposed to short laboratory experiments with amendments of NH_4_, NO_3_, orthophosphate, or a combination of all three. In three of four experiments, biomass responses were consistent with N‐limitation based on chlorophyll *a* or biovolume estimates. In experiments with evidence for N‐limited growth, MC gene expression was lower in NH_4_ treatments than in the control in 2021 but higher than in the control in 2022. The proportion of heterocytes (specialized cells for N fixation) was positively correlated with MC gene expression. These experiments reinforce the strong connection between N physiology and MC gene expression, but variation in taxonomic composition within genera or even species remains a possible cause of inconsistency in whole‐community responses.

**Abbreviations**
ANA: anatoxin
ANOVA: analysis of variance
BC: Bacillariophyta
BD: below detection limit
C: control
CH: Chlorophyta
Cl: chloride
CR: cryptophyte
*C*_T_
: Cycle threshold
CY: Cyanobacteria
CYN: cylindrospermopsin
DIN: Dissolved inorganic nitrogen
EB‐21: Eck Bay 2021
EL‐22: Ellsworth Bay 2022
HABS: harmful algal blooms
K: potassium
MC: microcystin
MD‐21: Maintenance dock 2021
MD‐22: Maintenance dock 2022
N: nitrogen
Na: sodium
NH_4_
: ammonium
NMDS: nonmetric multidimensional scaling
NO_3_
: nitrate
NO_X_
: nitrate plus nitrite
O: oxygen
OP: orthophosphate
P: phosphorus
qPCR: quantitative polymerase chain reaction
qRT‐PCR: quantitative reverse transcription polymerase chain reaction
SAX: saxitoxin
SRP: Soluble reactive phosphorus
TDN: Total dissolved nitrogen
TDP: Total dissolved phosphorus
TN: total nitrogen
TP: Total phosphorus
USGS: United States Geological Survey

## INTRODUCTION

Harmful algal blooms (HABs) are a significant threat to the health of lakes and their surrounding communities (Amorim & Moura, 2021; Smith & Schindler, 2009). In freshwater, most HABs are caused by cyanobacteria (Huisman et al., 2018). Cyanobacteria blooms cause harm by providing poor food resources to other trophic levels, by creating anoxia when they die and begin to decompose, and by producing toxins that are harmful to a wide variety of organisms (Bownik, 2016; DeMott & Muller‐Navarra, 1997; Larson et al., 2018; Wurtsbaugh et al., 2019). National and international agencies typically provide guidelines related specifically to the concentration of individual cyanotoxins, but those guidelines often lack quantitative estimates for identifying what constitutes a bloom (Chorus & Welker, 2021; United States Environmental Protection Agency, 2019). This lack is due to the variety of ways to define HABs (Gorney et al., 2023). In Kabetogama Lake, the system we studied, HABs are typically identified by the presence of visual indicators, and these blooms have been shown to contain cyanotoxins (Christensen et al., 2019; Minnesota Pollution Control Agency, 2025). Both growth and toxin production are involved in defining blooms and the associated harms.

As a result of the wide array of harms that cyanobacterial blooms can cause, it is necessary to evaluate the environmental drivers that allow blooms not only to grow and be sustained but also to trigger toxin production. Numerous environmental drivers have been linked to cyanotoxin production that are not necessarily related to the drivers of cyanobacterial growth (Omidi et al., 2018; Zurawell et al., 2005). The primary driver of HAB occurrence is nutrient pollution, a term usually including both phosphorus (P) and nitrogen (N; Heisler et al., 2008, Glibert, 2017). Bloom‐forming cyanobacteria grow most efficiently when supplied with inorganic reduced N (ammonium) and inorganic P (orthophosphate) compared with other sources of N and P (Inabe et al., 2021; Oliver et al., 2012). Increases in external loads of these forms of N and P in lakes and reservoirs are often associated with an increased likelihood of HAB occurrence (Heisler et al., 2008; Kotak et al., 2000). In addition to external loads, internal nutrient loading can contribute significantly to HAB occurrences, and often internal loads contribute more during the periods when blooms occur (Kowalczewska‐Madura et al., 2022; Matisoff et al., 2016; Orihel et al., 2017; Søndergaard et al., 2013). Lake stratification often occurs in deeper areas of lakes, possibly cutting off the surface waters from these sediment sources of nutrients, but in nearshore areas, sediment nutrient supply could be an important driver of the phytoplankton community.

There is a tendency to use statistical or process models to identify environmental or landscape drivers of chlorophyll *a* concentration or cyanobacterial biomass and infer that those biomass indices are sufficient to predict harms associated with cyanobacterial blooms (Janssen et al., 2019). These models do a decent job of predicting cyanotoxin concentrations at large spatio‐temporal scales because the presence of cyanobacteria is a necessary (though not sufficient) condition for cyanotoxins to be present (Graham et al., 2004; Yuan & Pollard, 2017). However, in field studies, the microcystin content per biovolume or per chlorophyll *a* is highly variable, and indices of biomass are often only weakly correlated to cyanotoxin concentrations (Catherine et al., 2008; Chaffin et al., 2021; Loftin et al., 2016). As a result, on a mechanistic level, the controls over cyanotoxin production are not necessarily the same factors that cause cyanobacteria to reach high abundance (Giblin et al., 2022; Omidi et al., 2018; Zurawell et al., 2005).

Many culture studies have demonstrated that cyanotoxin production varies according to environmental conditions and strain identity (Ginn et al., 2010; Jähnichen et al., 2011; Pimentel & Giani, 2014; Song et al., 1998; Yancey et al., 2023). Much of this research focused on *Microcystis* spp. and the microcystin (MC) cyanotoxin, which has been most observed cyanotoxin in surveys of lakes and rivers of North America (Graham et al., 2020; Loftin et al., 2016; Omidi et al., 2018). Microcystin is an N‐rich molecule (as are other cyanotoxins), and therefore many of the hypothesized drivers of MC production relate to the form and availability of N to cyanobacterial cells. Ginn et al. (2010) observed that reducing N in the media of a *Microcystis aeruginosa* strain resulted in the increased expression of genes related to both N stress (i.e., when either N is limiting or when preferred forms of N are absent) and MC production, consistent with some earlier culture studies (Ginn et al., 2009; Vezie et al., 2002). Zhou et al. (2020) also observed that N starvation resulted in increased expression of MC production genes and high MC content. These results are counterintuitive from the perspective of optimal resource use, because the limited available N could be used for primary metabolism and growth instead of producing a secondary metabolite (Chaffin et al., 2023; Van de Waal et al., 2009). Some studies have suggested that MC is a primary metabolite that assists in regulating metals when cyanobacteria produce enzymes requiring metal cofactors to use nitrate, urea or N_2_ (Alexova et al., 2011; Ceballos‐Laita et al., 2017; Omidi et al., 2018; Sevilla et al., 2008; Wagner et al., 2021). Microcystin could also have important functional roles during many kinds of stress beyond N stress, including during photo‐oxidation and temperature stresses (Meissner et al., 2015; Wei et al., 2024; Zilliges et al., 2011). Although these culture studies have suggested that additions of reduced inorganic N (NH_4_, the most preferred N form for most cyanobacteria) would probably decrease MC production (as it would alleviate N stress), there have also been studies that seem to indicate MC production is unaffected or even enhanced by the addition of NH_4_ (Horst et al., 2014; Krausfeldt et al., 2020).

Between landscape‐scale correlative analyses and single‐strain culture studies are microcosm or mesocosm studies that have attempted to measure the response of naturally occurring phytoplankton communities to short‐term changes in conditions. Davis et al. (2015) is an example of this type of study, in which communities in a Great Lakes embayment dominated by *Planktothrix* spp. were amended with P and/or one of several forms of N (NH_4_, nitrate, and urea). Contrary to the expectations derived from culture studies, MC appeared to be stimulated in this system by all forms of N. Conversely, a study of northern California reservoirs dominated by *Microcystis* determined that although growth was N‐limited, additions of N often had no effect on microcystin concentrations (Moisander et al., 2009). In other systems, the effects of nutrient additions on toxin production were shown to vary with time and community composition (Donald et al., 2011). Some authors have also speculated that during P limitation, cyanobacteria could even funnel extra N into microcystin and other N‐rich cyanotoxins as a part of N “overflow metabolism,” analogous to terrestrial plant production of carbon‐rich secondary metabolites when carbon is in excess (Glibert, 2017; Hill & Shlisel, 2024). Many studies on naturally occurring phytoplankton communities were short‐term, to minimize chamber effects while still allowing for growth (48–72 h; Lewis & Wurtsbaugh, 2008, Chaffin et al., 2014, Davis et al., 2015, Xu et al., 2015, Stoll et al., 2024).

The objective of this study was to identify the effect of nutrients on growth and toxicity of naturally occurring cyanobacterial communities in Kabetogama Lake using experimental microcosms. We hypothesized that short‐term nutrient limitation on growth will interact with cyanotoxin production. Specifically, we hypothesized that MC production is strongly influenced by the availability and form of N, depending on whether the community is growth‐limited by N or P. We hypothesized that during P‐limitation, cyanobacteria are receiving excess N that could be used in the production of MC and other N‐rich cyanotoxins, a form of “overflow metabolism” (Glibert, 2017; Glibert & Burkholder, 2011). In this scenario, additions of N would increase toxin production, and increases of P would reduce the amount of excess N and, therefore, decrease toxin production. In contrast, during N‐limitation or N‐stress (i.e., when reduced inorganic N is unavailable), cyanobacterial cells would activate alternative N‐acquisition pathways to use nitrate, atmospheric N_2_ and organic forms of N (Oliver et al., 2012), which trigger production of MC. We hypothesized that N‐limited communities would decrease MC production when ammonium (NH_4_) is provided. Microcystin production is difficult to measure directly over short time intervals (hours to days) because MC often degrades slowly (over days to weeks), although this can be highly variable depending on the ambient bacterial community (Dziga et al., 2017; Edwards et al., 2008). Therefore, we monitored MC production by measuring the RNA copies of a gene involved in MC synthesis (*mcy*E), which probably has a decomposition half‐life of minutes or hours (Steiner et al., 2019; Zhang et al., 2022). As a result, increases and decreases in expression of the *mcy*E gene are a much more sensitive indicator of the cyanobacteria's attempts to produce MC. At the same time, we monitored the response of genes associated with three other cyanotoxins (cylindrospermopsin, anatoxin, and saxitoxin), about which less is known.

## MATERIALS AND METHODS

### Study sites

Kabetogama Lake is part of the Namakan‐Rainy reservoir system, located on the border between the United States and Canada. A hydroelectric dam at the outflow of Rainy Lake is used to provide power for surrounding communities, and water levels are managed at the outflow of Namakan Lake to support management of Rainy Lake water levels (Kallemeyn et al., 2003). Kabetogama Lake drains into Namakan Lake (except at higher water levels when Kabetogama Lake drains directly into Rainy Lake). Kabetogama Lake is polymictic, with a mean depth of 9 m and a maximum depth of 24 m. Kabetogama Lake is often considered to be eutrophic (Christensen & Maki, 2015), and during the late summer and fall, cyanobacterial HABs are periodically observed within nearshore areas and embayments. The bloom‐forming taxa in Kabetogama Lake include several Cyanobacterial genera: *Dolichospermum*, *Aphanizomenon*, *Planktothrix*, and *Pseudanabaena*, among others (Christensen et al., 2019). During blooms, several cyanotoxins have been detected (e.g., MCs; anatoxin‐a, ANA; and saxitoxin, SAX), occasionally at relatively high concentrations (Christensen et al., 2019). These cyanotoxins have many congeners that vary in toxicity and other properties, but they have been lumped together here for convenience (Metcalf & Codd, 2012). Anoxia has been observed in deeper areas of the lake, including in embayments with depths >4 m (Christensen et al., 2013).

**FIGURE 1:**
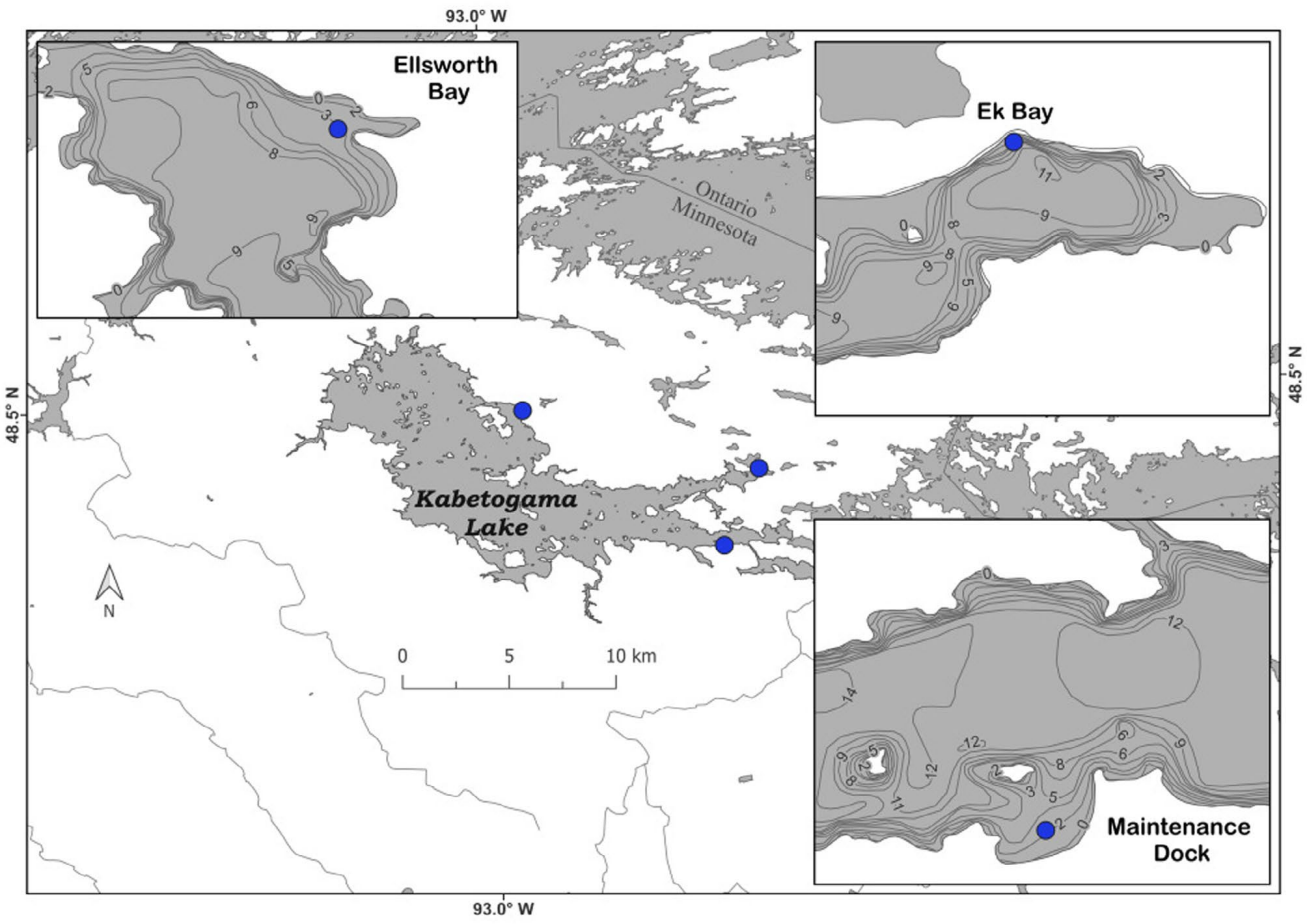
Locations from which initial phytoplankton communities were collected during September 2021 and 2022 from Kabetogama Lake (Minnesota, United States). Contour lines are shown with depth in meters from the maximum water level elevation. Ek Bay and the Maintenance Dock sites were sampled in 2021; Ellsworth Bay and the Maintenance Dock sites were sampled in 2022.

Environmental controls on the distribution and occurrence of blooms in Kabetogama Lake are not well understood, but blooms tend to occur late in the growing season (August–October). Our experiments occurred in mid‐September, and we selected sampling locations based on visible surface conditions in the 1–7 days prior to sampling. Three locations were sampled: (1) a site near the Voyageurs National Park Ash River maintenance dock (2021 and 2022) near U.S. Geological Survey (USGS) site 482603092511801 (referred to as Maintenance Dock hereafter), (2) Ek Bay (2021 only) near USGS site 482804092494701 and (3) Ellsworth Bay (2022 only) near USGS site 482947092584401 (Figure 1). These sites all had reports of visible surface scums during midday and visual cues suggesting cyanobacterial colonies (Rosen & St. Amand, 2015). All sites are relatively shallow embayments in the nearshore zone.

At each site, at about 8 a.m. local time on the day of the experimental setup, water was pumped from approximately 1 m below the surface into a plastic 60‐L tub, which was continuously stirred. From this plastic tub, 40 L of water was collected into two 20‐L containers for transport back to the laboratory for the incubation experiments.

### Laboratory experiments

Field water was returned to the lab following collection for the laboratory incubation experiment. At the lab, both containers from a single site were combined into a single tub that was kept well mixed. Triplicate samples were collected for nutrients, chlorophyll *a*, and phytoplankton community composition. Two samples were collected to measure DNA copies of toxin production genes, and one sample was collected to measure RNA copies of these genes. At the same time, borosilicate bottles were filled with 1 L of homogenized lake water and randomly assigned a treatment combination. Treatments were created by adding stock solutions of P (K_2_HPO_4_; 1550 mg P · L^−1^), NH_4_ (NH_4_Cl; 2800 mg N · L^−1^), and/or NO_3_ (NaNO_3_; 2800 mg N · L^−1^) to each chamber. To each chamber in the P treatment, we added 1 mL of the P stock solution plus 2 mL of filtered water. To each chamber in the NH_4_ treatment, we added 1 mL of the NH_4_ stock plus 2 mL of filtered water. To each chamber in the NO_3_ treatment, we added 1 mL of the NO_3_ stock plus 2 mL of filtered water. To create the N + P treatment, we added 1 mL from each stock solution (P, NH_4_, and NO_3_). The target concentration for P was therefore 1.55 mg P · L^−1^, consistent with concentrations in COMBO media that is intended to alleviate nutrient limitation (Kilham et al., 1998). We set target concentrations for N (as either NO_3_ or NH_4_) at a value we believed would be well above what we would expect to see in field conditions (2.8 mg N · L^−1^), based on observational data from earlier sediment flux studies and our previous phytoplankton enrichment studies (Larson et al., 2023; Larson, Bailey, Maki, et al., 2025; Larson, Bailey, & Stelzer, 2025). Because we could not measure ambient nutrients at the time of experimental setup, this did not take into account ambient concentrations of N or P. After accounting for ambient N and P concentrations, TN:TP molar ratios in the +P treatment were 1.0, 1.1, 0.8, and 0.8 in the Ek Bay (2021), Maintenance Dock (2021), Ellsworth Bay (2022), and Maintenance Dock (2022) experiments, respectively. The TN:TP molar ratios in the +NH_4_ or + NO_3_ treatments were 270.6, 167.9, 214.7, and 202.5 (same experiment order) and in the treatment with N and P amendments the TN:TP molar ratio were 8.9, 8.8, 8.6, and 8.6 (again, in the same experiment order).

After bottles were filled and spiked with nutrient treatments, they were inverted several times and placed in an incubation chamber set to the lake water temperature at the time of collection. Light was provided on a 12:12 day‐night cycle (consistent with the day‐night interval for September at Kabetogama Lake) by a bank of LED grow lamps (Durolux DLED824W LED Grow Light; 5812 lumens output). Treatments were arranged in a stratified random pattern from front to back to minimize the effect of location within the experimental chamber on potential treatment effects. Each day, bottles were mixed by inversion at least twice (at approximately 8 a.m. and 5 p.m.), but there were particles that settled between inversions. Between inversions, bottles were capped loosely. No noticeable biofilms formed in the bottles. After 48 h, starting at approximately midday (i.e., the middle of the light period in the chambers), each bottle was thoroughly mixed and samples were collected for phytoplankton community composition (120 mL), chlorophyll *a* (100–200 mL) and quantification of RNA copies of cyanotoxin genes (50–60 mL). The exact volume sampled and the time at which sampling was started were recorded in Larson, Bailey, Maki, et al. (2025); Larson, Bailey, & Stelzer (2025).

### Nutrient analysis

Nutrient samples were processed following methods described in Soballe and Fischer (2004) at the Upper Midwest Environmental Sciences Center Water Quality Lab. Collection methods were identical to those previously reported in other studies (Larson et al., 2019). Whole water samples for total N (TN) and total P (TP) were acidified and then refrigerated until analysis. Dissolved nutrients were filtered (0.45‐μm) and either acidified and refrigerated in the case of total dissolved P (TDP), total dissolved N (TDN), nitrate+nitrite (NO_X_) and ammonium (NH_4_^+^) or frozen in the case of soluble reactive P (SRP). Method detection limits reported by the Water Quality Lab were: 0.001 mg · L^−1^ SRP, 0.001 mg · L^−1^ TP/TDP, 0.01 mg · L^−1^ nitrate plus nitrite N (NO_X_), 0.008 mg ·L^−1^ ammonium N (NH_4_), and 0.01 mg · L^−1^ TN/TDN (Soballe & Fischer, 2004). When calculating nutrient ratios, we used NH_4_:SRP ratios instead of dissolved inorganic N to SRP ratios (DIN:SRP), because almost all of our samples had concentrations of NO_X_ below the detection limit.

### Chlorophyll *a*

Chlorophyll *a* concentrations were measured on initial water samples and after experimental treatments. Between 100 and 500 mL of water was filtered onto 1‐μm glass fiber filters (Pall 61631) that were held frozen until they were processed (100–200 mL for experimental treatments; 250–500 mL in samples from the initial water source). Chlorophyll *a* was determined spectrophotometrically (American Public Health Association [APHA], 2012) following extraction in acetone (Soballe & Fischer, 2004). Filtering for chlorophyll *a* took place outside of direct sunlight, and chlorophyll *a* samples were processed within 30 days of collection.

### Phytoplankton community composition

Approximately 120 mL of whole water from experimental chambers and initial source water was collected for phytoplankton community composition and was preserved with 3 mL Lugol's iodine solution (Intergovernmental Oceanographic Commission, 2010). Samples were analyzed for phytoplankton abundance and community composition by membrane filtration and microscopy by BSA Environmental, Inc. (Cleveland, Ohio, United States). Cell numbers were quantified using the Utermöhl method (APHA, 2012) using a 300‐natural unit threshold or 50 random fields for counts. Aliquots were placed into custom Utermöhl chambers and allowed to settle in darkness undisturbed for at least 20 h within an enclosure protected from vibration and temperature alteration. Ten specimens of each taxon in each sample were measured to obtain biovolume estimates. Cell biovolumes of all identified phytoplankton taxa were estimated using formulae for solid geometric shapes that most closely matched the cell shapes (Hillebrand et al., 1999). Natural units were defined as a group of attached cells (e.g., colonies of *Microcystis* or *Dolichospermum*) or an individual cell. The exception was that each diatom cell was considered a natural unit, even if they were in a chain. Taxonomy follows nomenclature according to AlgaeBase (Guiry & Guiry, 2026; nomenclature accurate as of January 2023).

In addition to preserving samples for microscopic examination of phytoplankton community composition, we also visually inspected samples using an imaging flow cytometer (the FlowCAM). The FlowCAM is a useful tool for characterizing particles and can be used for rapidly estimating biovolumes and community composition in phytoplankton (Hrycik et al., 2019; Milde et al., 2017; Owen et al., 2022). In this case, we used the images to observe whether cyanobacterial colonies had heterocytes or not (Chaffin et al., 2020). For all treatments in 2021 and in initial water samples from both 2021 and 2022, we looked at the 25 largest images of *Aphanizomenon* and *Dolichospermum* colonies and counted the number that had visible heterocytes (see examples in Figure S1). We chose these taxa because they are usually very easy to see in the FlowCAM images, and the heterocytes are often very clearly visible (Chaffin et al., 2020). In a few samples, only 20–24 images were available. In 2022, we looked at initial water samples, but when we attempted to collect images at the end of the experiment, we encountered difficulties with the FlowCAM and were not able to generate images from most experimental chambers. Therefore, only the effects of nutrient amendments in 2021 on heterocyte proportion could be estimated. No other heterocyte‐producing taxa were observed in the FlowCAM images. Microscopy did identify *Raphidiopsis*, which can produce heterocytes, in samples, but they were a small part of the community in 2021 when we collected most FlowCAM images.

### DNA and RNA cyanotoxin gene analysis

Samples for cyanotoxin synthetase gene analysis were collected by filtering up to 100 mL of sample water onto a 0.2‐μm polycarbonate filter (Isopore GTTP02500) using sterile components (filter funnels were decontaminated using a bleach rinse between uses). Filters were then folded using sterile forceps into bead tubes (0.3 g of acid‐washed glass beads in a 1.5‐mL screw‐cap centrifuge tube) and immediately frozen using liquid nitrogen. Bead tubes were prepared in advance by the USGS Ohio Water Microbiology Laboratory. Frozen samples were sent to the USGS Ohio Water Microbiology Laboratory on dry ice, where quantitative polymerase chain reaction (qPCR) and quantitative reverse transcription PCR (qRT‐PCR) were used to quantify DNA concentration and RNA expression of microcystin synthetase (*mcy*E) gene copies, anatoxin‐a synthetase (*ana*C) gene copies, cylindrospermopsin synthetase (*cyr*A) gene copies, and saxitoxin synthetase (*sxt*A) gene copies (Al‐Tebrineh et al., 2012; Sabart et al., 2015).

Filters for DNA analysis were extracted using a DNA‐EZ extraction kit (GeneRite, North Brunswick, New Jersey, United States) according to the manufacturer's instructions except that no prefilter was used; filters for RNA analysis were extracted using a RNeasy PowerPlant extraction kit (Qiagen LLC, Germantown, Maryland, United States) according to the manufacturer's instructions with the addition of a DNase treatment. All assays were run on either an Applied Biosystems™ (Waltham, Massachusetts, United States) StepOnePlus™ or a QuantStudio™ 3 thermal cycler. Primer and probe sequences and run conditions for each assay are listed in Table S1. Depending on the assay, either TaqMan™ Universal PCR Master Mix or SYBR™ Green Universal PCR Master Mix (Applied Biosystems™, Waltham, Massachusetts, United States) was used. Extracted RNA was first checked for complete DNA degradation and then reverse transcribed to complementary DNA (cDNA) as a two‐step process before qPCR analysis using the same assays listed in Table S1.

Plasmid standards for each assay were used to establish standard curves for quantification. The copy number of each plasmid standard was calculated using the DNA concentration measured by the Qubit dsDNA High Sensitivity Assay (Life Technologies, Carlsbad, California, United States) and the molecular weight of the plasmid. Standard curve characteristics and limits of detection for each assay are supplied in Table S2. Sample inhibition was determined via matrix spikes. For DNA samples, a duplicate master mix was seeded with target DNA, whereas for RNA samples, the reverse transcription reaction was seeded with armored RNA. If the seeded test sample was more than 2 cycle threshold (*C*_T_) higher than the seeded clean matrix control, the sample was diluted and rerun.

Quality control samples for all qPCR and qRT‐PCR assays included process blanks (deionized water filtered instead of sample water), extraction blanks, qPCR/qRT‐PCR blanks, and positive control standards. All blank results showed no detection. Samples were stored at −80°C until analysis (within 4 months for 2021 samples and within 3 months for 2022 samples). In addition to measuring *mcy*E RNA gene copies for each individual chamber at the end of the experiment, we also calculated *mcy*E RNA gene copies per cyanobacterial biovolume in the same chamber.

### Statistical analysis

All statistical analyses were performed in R Version 3.6.1 (R Development Core Team, 2022). Standard descriptive statistics were estimated using base R functions in most cases. All analyses were done for each experiment separately. When samples included non‐detects, we used Kaplan–Meier methods for estimating mean, median, and standard deviation. Kaplan–Meier survival analysis is a non‐parametric method for estimating descriptive statistics when data are right‐censored and is recommended to use in reverse format to handle data with non‐detects (Helsel, 2005; Julian & Helsel, 2021; Lee, 2020). We used a Bayesian analysis of variance (ANOVA) to identify effect sizes between control treatments and nutrient treatments for chlorophyll *a* concentration, total phytoplankton biovolume, proportion of images with heterocytes, Bacillariophyta biovolume, Chlorophyta biovolume, Cyanobacteria biovolume, *Aphanizomenon* biovolume, and RNA copies of *mcy*E (normalized by biovolume or concentration in water; Kelter, 2022). In this approach, the data are checked for normality using the Shapiro–Wilk test, and then (if appropriate) a normal distribution is estimated for each treatment using the experimental data. Other phytoplankton genera could not be analyzed with this method because of either a high number of non‐detects or non‐normally distributed data. In our case, all analyses were natural log transformed, so this was effectively a log‐normal distribution. After estimating the distributions using the data, the distributions were sampled 10,000 times, and a distribution was estimated for the differences between the treatments. When the 95% credible interval of the distribution of differences between two treatments did not overlap zero, we considered these treatments to be distinct (Kelter, 2022; McCarthy, 2007).

To estimate differences in the biovolume in all other taxa among treatments, as well as differences in *ana*C, *cyr*A, and *sxt*A RNA gene copies, we used a “flipped” non‐parametric survival analysis, due to non‐detects (Helsel, 2005, 2011; Lee, 2020). The version of this analysis used was the Peto‐Peto test, implemented with the NADA and survminer packages in R (Helsel, 2011; Kassambara et al., 2021; Lee, 2020). All of the Peto‐Peto comparisons are between a treatment and the control and, therefore, have 1 degree of freedom. Differences between treatment and control that met the arbitrary threshold of *p* < 0.05 (or a chi‐squared value of >3.84) were considered distinct.

To visualize among‐treatment differences in community composition, we plotted samples from each experiment in two‐dimensional space using nonmetric multidimensional scaling (NMDS) and then drew ellipses around each treatment with the 95% standard error around the center of the treatment observations (Borcard et al., 2011) using the vegan package in R (Dixon, 2003).

## RESULTS

### Initial conditions

**FIGURE 2:**
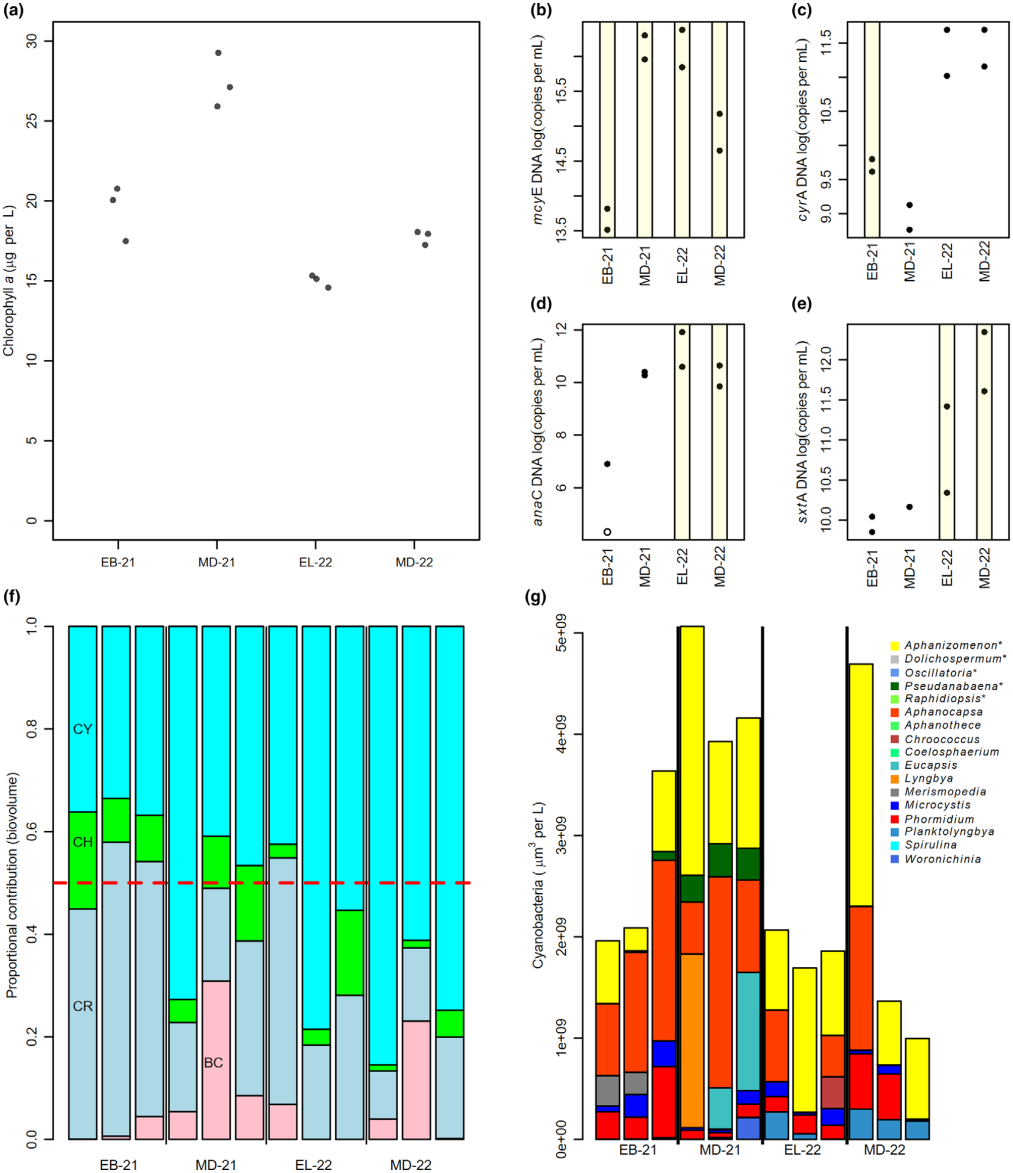
Initial conditions of the phytoplankton community collected from Kabetogama Lake (Minnesota, United States) in 2021 and 2022. These are initial conditions from four experiments: EB‐21 (Ek Bay in 2021), MD‐21 (Maintenance Dock in 2021), EL‐22 (Ellsworth Bay in 2022) and MD‐22 (Maintenance Dock in 2022). (a) Measured chlorophyll *a* in lake water at the beginning of each experiment. (b–e) Concentrations of cyanotoxin synthetase genes. Dots indicate the log of the DNA gene copies per mL. Filled circles indicate values above the detection limit; open circles indicate values below the detection limit. Vertical yellow lines indicate RNA expression of that gene was found above the detection limit. *ana*C, anatoxin; *cyr*A, cylindrospermopsin; *mcy*E, microcystin; *sxt*A, saxitoxin. (f) Average relative contribution of cyanobacteria (CY), chlorophyta (CH), cryptophyta (CR) and bacillariophyta (BC) to the total biovolume. (g) Average total biovolume of all cyanobacterial genera in each initial sample (only genera with a median > 0 are shown). * indicates genera with known N‐fixing species.

The sites sampled for these experiments all had visual indicators of surface blooms within the week prior to the start of the experiment (surface scums and green water). At the start of the experiments, chlorophyll *a* ranged from 15 to 30 μg · L^−1^ (Figure 2a), and DNA for all four of the cyanotoxin classes measured was present in the community (Figure 2b–e). Whereas we consider DNA to be indicative of the potential for toxin production, the presence of RNA indicates cyanobacteria are expressing the relevant genes. In lake water at the start of all experiments, microcystin (*mcy*E) RNA gene copies were above the detection limit (Figure 2b, yellow bands). Cylindrospermopsin (*cyr*A) RNA gene copies were only detected at the Ek Bay site in 2021 (Figure 2c), and anatoxin (*ana*C) and saxitoxin (*sxt*A) RNA gene copies were above the detection limit at both sites sampled in 2022 (Figure 2d,e). Cyanobacteria made up a large fraction of the initial community in each experiment (Figure 2f), but the Cyanobacteria present varied considerably among sites and years (Figure 2g). The Ek Bay community included more *Dolichospermum* than other Cyanobacteria but also included *Aphanizomenon* and *Pseudanabaena* (Figure 2g). These taxa also appeared in the Maintenance Dock site in 2021, along with *Microcystis*, *Oscillatoria*, and *Chroococcus* (Figure 2g). In 2022, *Aphanizomenon* was the most common taxa in almost all samples, with *Dolichospermum* and *Raphidiopsis* usually making up smaller fractions (Figure 2g). Other Cyanobacterial genera that appeared with a median biovolume >0 are listed in Figure 2g. Heterocytes were visible in 16%–32% of *Aphanizomenon* colonies and 15%–42% of *Dolichospermum* colonies at the start of the experiments (Table 1), with higher proportions at the Maintenance Dock site in both years.

**TABLE 1:** Proportion of colonies observed using a flow cytometer that contained visible heterocytes in samples from the phytoplankton community in the initial water samples for each experiment.

Site	*Aphanizomenon*	*Dolichospermum*
Ek Bay (2021)	0.16	0.15
Maintenance Dock (2021)	0.23	0.28
Ellsworth Bay (2022)	0.16	0.22
Maintenance Dock (2022)	0.32	0.42

*Note*: In 2021, the proportion was the average from three samples, whereas in 2022, only one sample for each site was measured. At least 20 images for each taxa were present in each sample. See examples of images for these taxa with and without heterocytes in Figure S1.

Nitrate plus nitrate (NO_X_) was below the detection limit in most of our surface water samples (Table 2). Nutrient ratios in the surface waters were mixed in terms of indicating potential nutrient limitation. Dissolved N and P were present, but in the case of N, most of the dissolved N (TDN) was not in the inorganic forms NH_4_ or NO_X_ (Table 2). Molar ratios of TN:TP were relatively high (35.5–56.6) compared to published thresholds indicative of co‐N and P limitation or possibly P‐limitation (co‐limitation when TN:TP = 20–50, with higher values indicating P limitation; Guildford & Hecky, 2000). In contrast, NH_4_ to SRP ratios were lower (8.2–39.0; Table 2).

**TABLE 2:** Initial water quality conditions at sites where naturally occurring phytoplankton community were obtained.

Site	TN	TP	TDN	TDP	NH_4_	NO_X_	SRP	TN:TP	TDN:TDP	NH_4_:SRP
Ek Bay (2021)	0.75	0.029	0.51	0.003	0.040	BD	0.002	56.6	331.7	39.0
Maintenance Dock (2021)	0.77	0.047	0.46	0.008	0.028	0.049^a^	0.005	35.8	126.0	11.4
Ellsworth Bay (2022)	0.60	0.035	0.42	0.011	0.036	BD	0.009	38.3	81.8	9.1
Maintenance Dock (2022)	0.59	0.037	0.40	0.008	0.020	BD	0.005	35.5	105.0	8.2

*Note*: All concentrations are means in mg · L^−1^. Nutrient ratios are molar with reported medians.

Abbreviations: BD, below detection limit; NH_4_, ammonium; NO_X_, nitrate + nitrite; SRP, soluble reactive phosphorus; TDN, total dissolved nitrogen; TDP, total dissolved phosphorus; TN, Total nitrogen; TP, total phosphorus.

^a^ Estimated using Kaplan–Meier methods due to one value being a non‐detect.

### Effects of nutrient addition on growth and community composition

**FIGURE 3:**
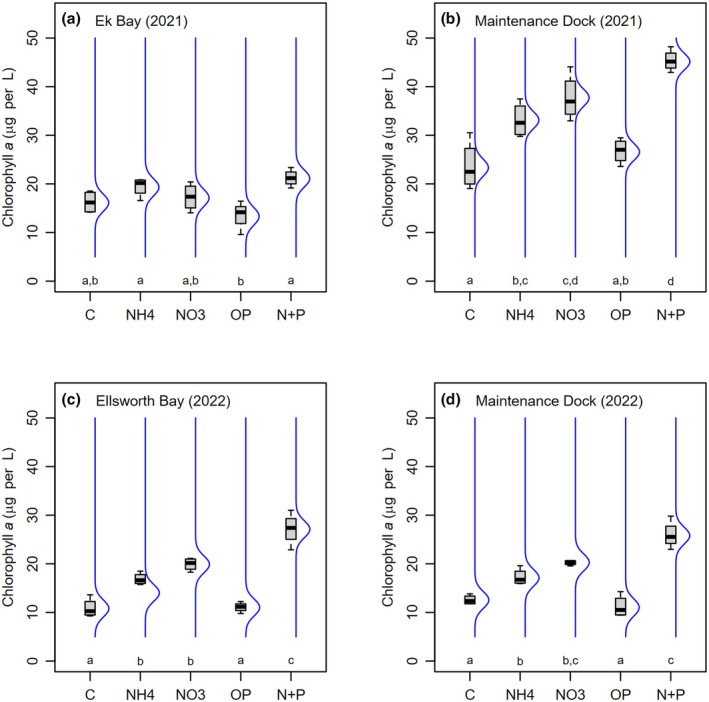
Box and whisker plot of chlorophyll *a* concentration of phytoplankton communities from Kabetogama Lake (Minnesota, United States) after experimental amendment at each site (four replicates per treatment). Blue lines indicate model distributions for each treatment. Letters indicate treatments that have overlapping distributions (i.e., differences between treatments that are not different from zero). C, Control; NH_4_, ammonium amendment; NO_3_, nitrate amendment; N + P, NH_4_ + NO_3_ + P amendment; P, orthophosphate amendment. Boxes encompass the first and third quartiles. The thick black line is the median. The lines (whiskers) show the largest or smallest observation that falls within 1.5 times the box size.

**FIGURE 4:**
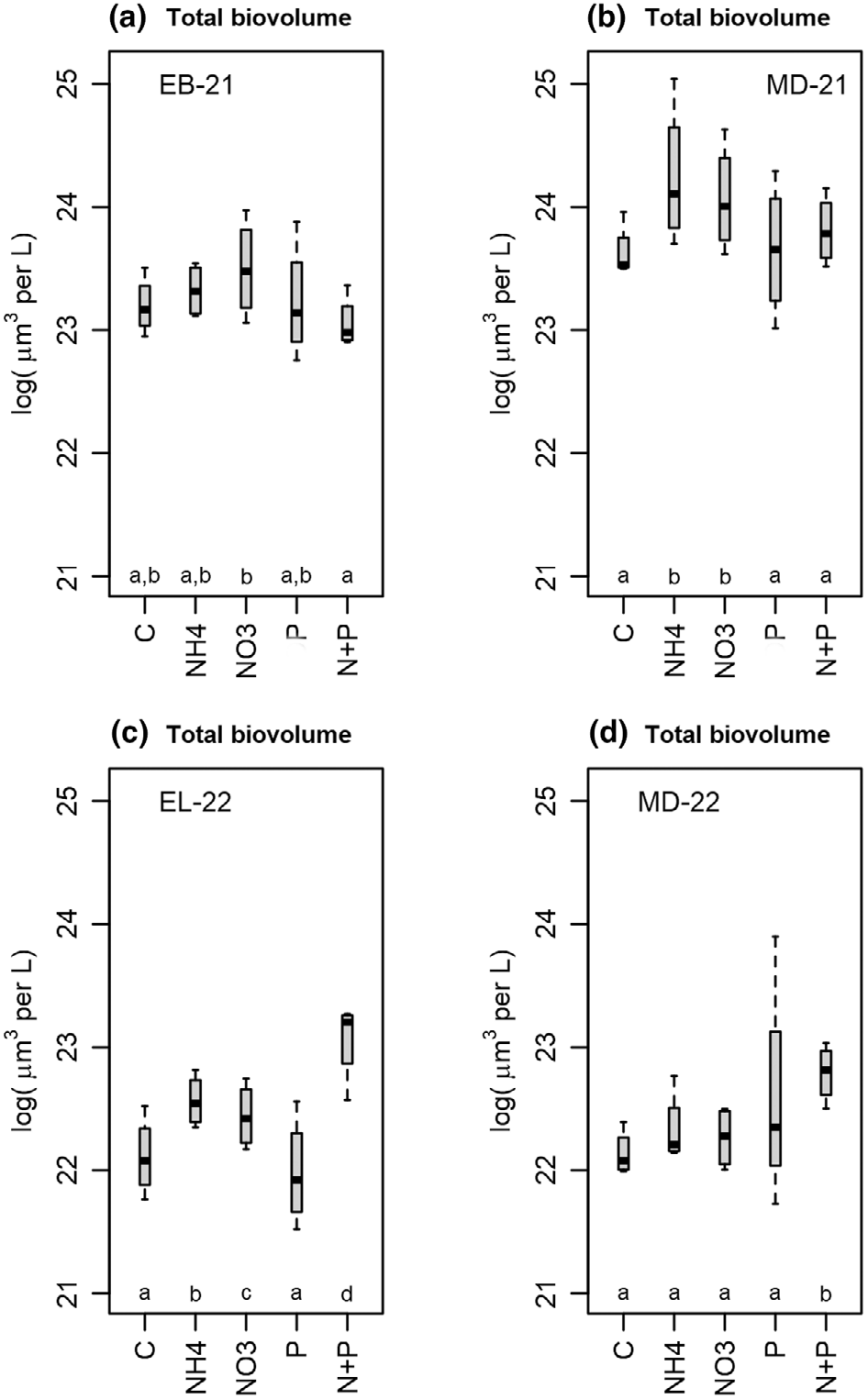
Box and whisker plot of total biovolume of phytoplankton communities from Kabetogama Lake (Minnesota, United States) after experimental amendment at each site (four replicates per treatment). Letters indicate treatments that have overlapping distributions (i.e., differences between treatments that are not different from zero). C, Control; NH_4_, ammonium amendment; NO_3_, nitrate amendment; N + P, NH_4_ + NO_3_ + P amendment; P, orthophosphate amendment. Boxes encompass the first and third quartiles. The thick black line is the median. The lines (whiskers) show the largest or smallest observation that falls within 1.5 times the box size.

**FIGURE 5:**
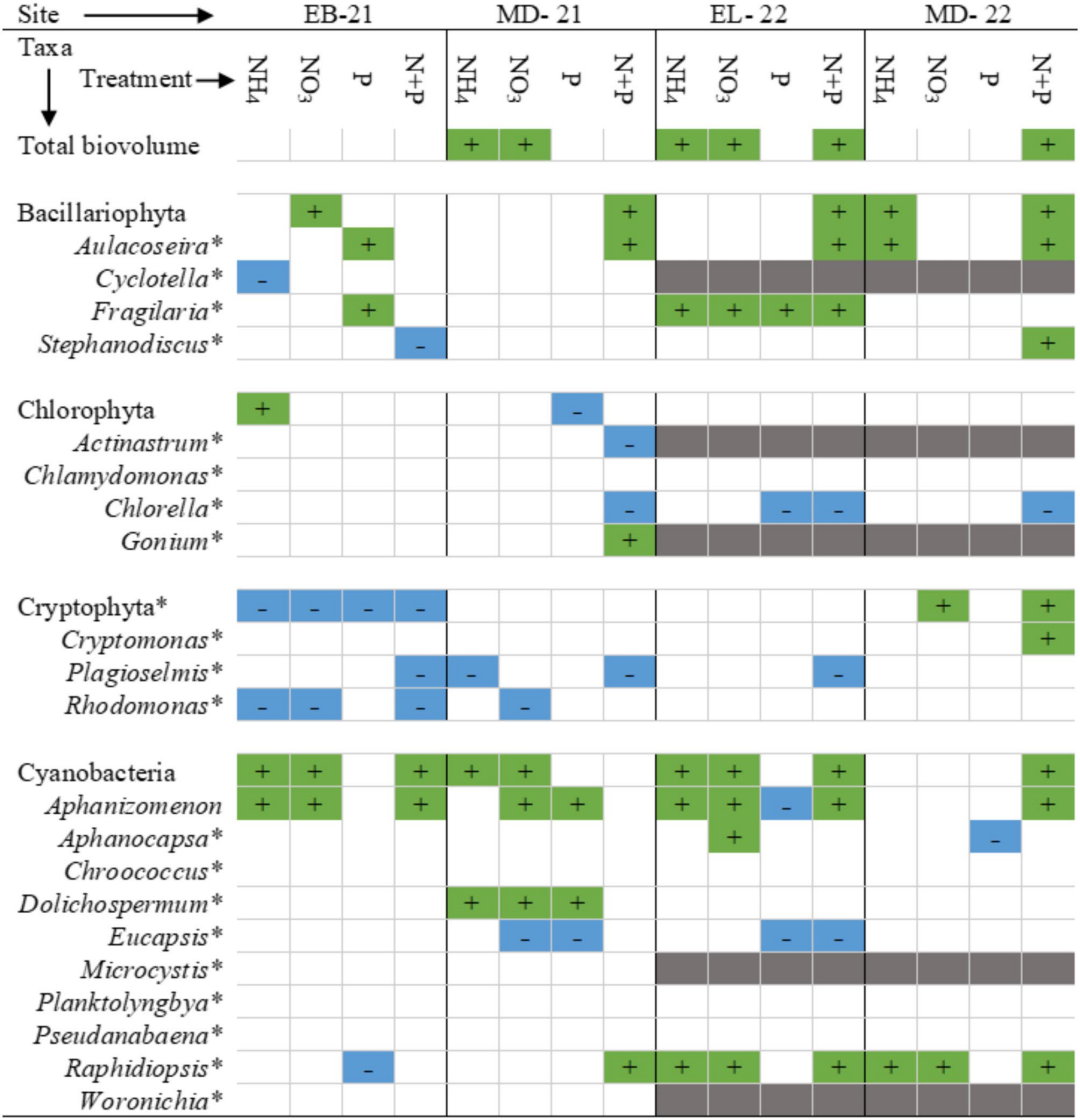
Response of biovolume concentration for total biovolume, phytoplankton divisions and individual genera from Lake Kabetogama (Minnesota, United States) during 2021 and 2022 to nutrient amendments relative to the control treatment. * indicates a non‐parametric Peto‐Peto test was used to assess differences from the control. Otherwise differences were assessed using Bayesian ANOVA. Green shading indicates positive effect (+), blue shading indicates negative effect (−), light gray shading indicates that taxa did not occur in any treatments. If there was no clear effect, the cell was not shaded.

**FIGURE 6:**
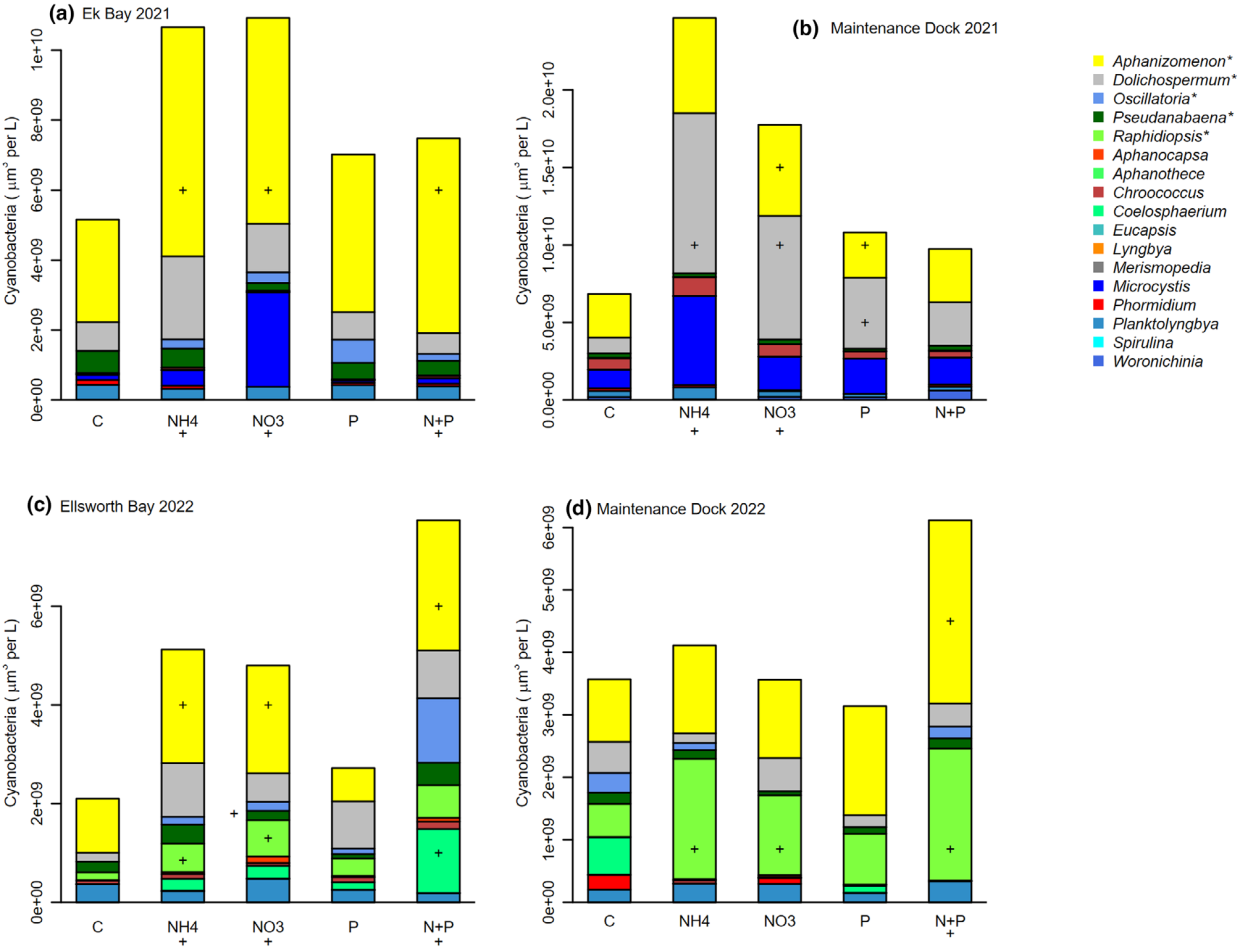
Average cyanobacterial biovolume of communities from Kabetogama Lake (Minnesota, United States) after experimental amendments at each site. Plus sign (+) indicates a higher biovolume relative to the control in either a specific genera or for the cyanobacteria as a whole (+ symbols below the treatment identification). Details of treatment effects provided in more detail in Table 3, as some genera cannot be visualized in this figure. C, Control; NH_4_, ammonium amendment; NO_3_, nitrate amendment; N + P, NH_4_ + NO_3_ + P amendment P, orthophosphate amendment. * indicates genera with known N‐fixing species.

**FIGURE 7:**
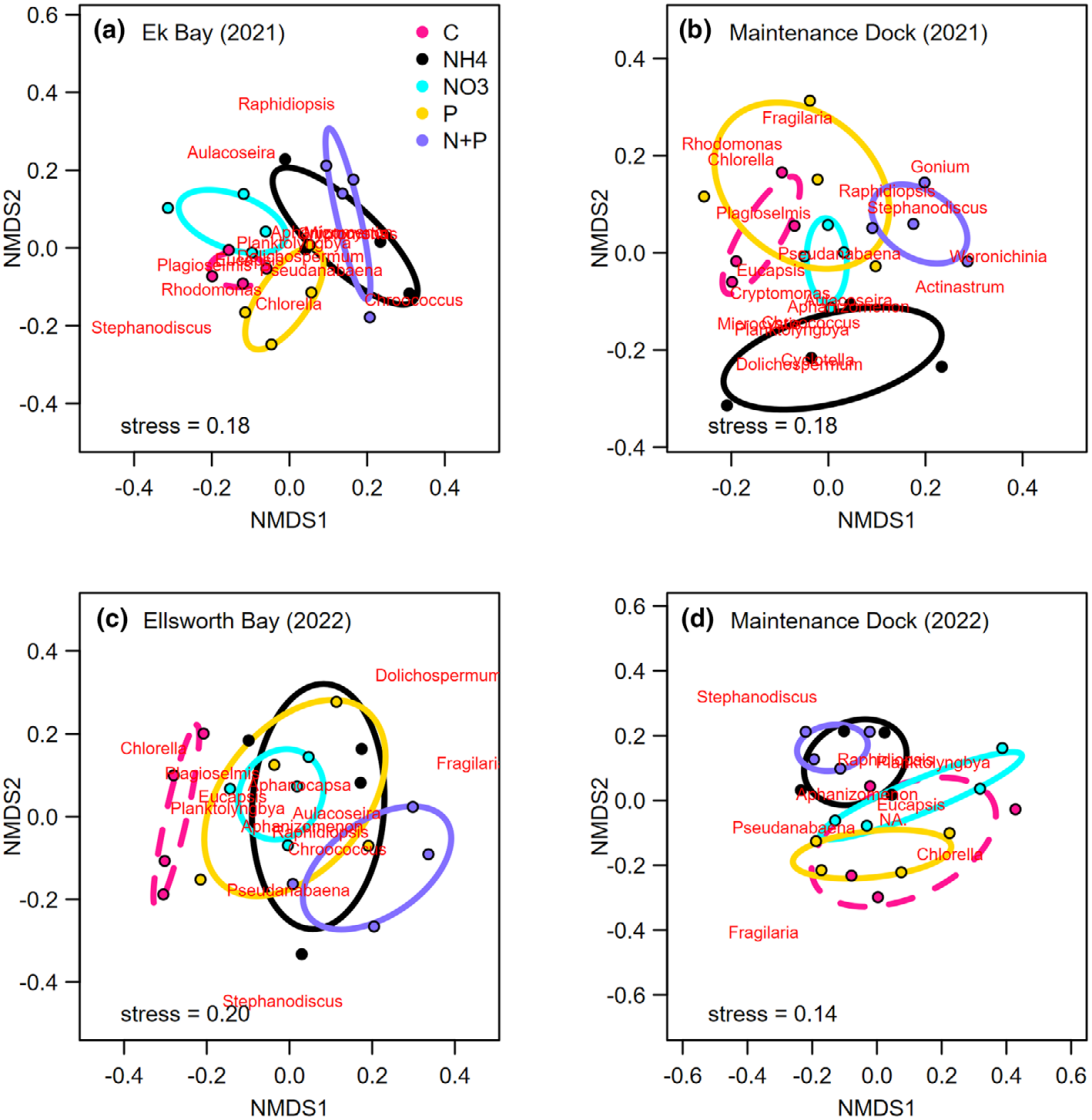
Nonmetric multidimensional scaling (NMDS) plot of phytoplankton communities from Kabetogama Lake (Minnesota, United States) after nutrient amendment experiment. Ellipses indicate the 95% standard error around the mean of samples from each treatment. Red text identifies the position of individual genera that occurred in that community with median biovolume > 0. Communities from individual replicates are indicated by dots. Two‐dimensional stress is reported. C, Control; NH_4_, ammonium amendment; NO_3_, nitrate amendment; N + P, NH_4_ + NO_3_ + P amendment; P, orthophosphate amendment.

In three of four experiments, chlorophyll *a* concentrations were elevated in treatments with N compared to control treatments (all but Ek Bay in 2021; Figure 3), and chlorophyll *a* was never higher in the P treatment than in the control. In the two 2022 experiments, it appeared that serial co‐limitation could have been occurring, as N + P treatments were higher than N‐alone treatments (Figure 3). Similarly, estimates of total community biovolume were higher than the control in either NO_3_ or NH_4_ in the 2021 Maintenance Dock experiment and the Ellsworth Bay experiment (2022; Figures 4 and 5). Experiments in 2022 indicated N + P co‐limitation (Figure 4). Biovolume of just cyanobacteria followed a similar pattern of N stimulation, with three of four experiments having higher values than the controls (Figure 5). These trends were driven by elevated biovolumes in *Aphanizomenon* and *Dolichospermum* in 2021 experiments and by *Aphanizomenon* and *Raphidiopsis* in 2022 experiments (Figures 5 and 6). The results of NMDS plotting indicated that communities from control treatments and NH_4_‐amended treatments had no overlap in three of four experiments (all but the Maintenance Dock 2022 experiment), and N + P treatments never fell within the 95% confidence interval ellipse for the control (Figure 7). Community composition within replicates experiencing NO_3_ or P amendments sometimes overlapped and sometimes did not overlap with control ellipses.

**FIGURE 8:**
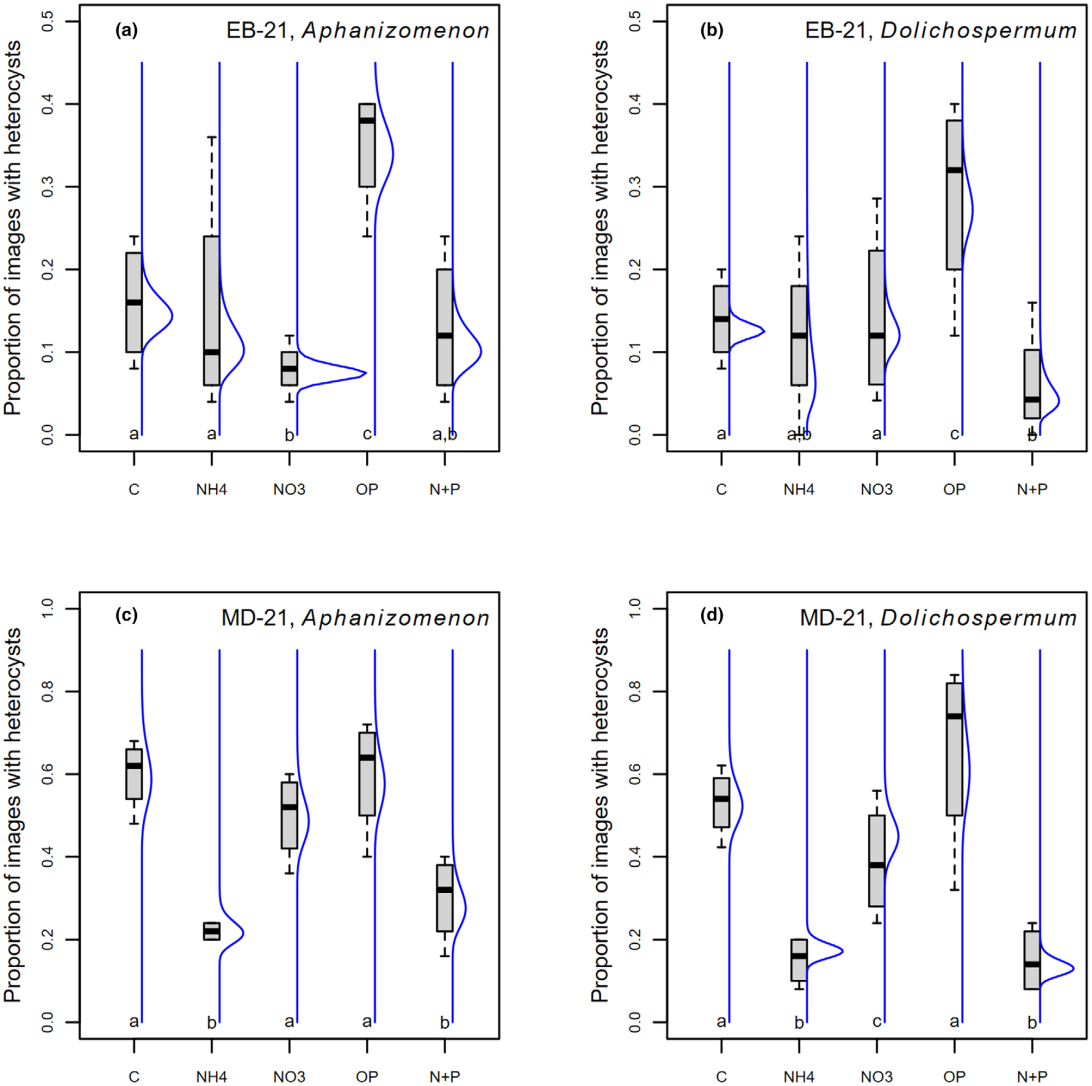
Box and whisker plot of the proportion of cyanobacterial colonies that had visible heterocytes in phytoplankton communities from Kabetogama Lake (Minnesota, United States) after experimental amendment at each site. Only *Aphanizomenon* and *Dolichospermum* colonies were visible and abundant enough to estimate proportions (see Figure S1 for examples). Blue lines indicate model distributions for each treatment. Letters indicate treatments that have overlapping distributions (i.e., differences between treatments that are not different from zero). C, Control; NH_4_, ammonium amendment; NO_3_, nitrate amendment; N + P, NH_4_ + NO_3_ + P amendment; P, orthophosphate amendment. Boxes encompass the first and third quartiles. The thick black line is the median. The lines (whiskers) show the largest or smallest observation that falls within 1.5 times the box size.

In the 2021 experiments, there was a very strong correlation between heterocyte presence in *Aphanizomenon* and *Dolichospermum* (Pearson's *r* = 0.71; 95% confidence interval 0.51, 0.83). In the Ek Bay experiment during 2021, heterocyte proportion in *Aphanizomenon* and *Dolichospermum* was higher in the P treatment than the control treatment, presumably due to P additions exacerbating N limitation (Figure 8). In Ek Bay, the control treatment had a heterocyte proportion that was similar to what was observed in the initial surface water (~15%, Table 1, Figure 8), but initial surface water from the Maintenance Dock in 2021 had a much lower heterocyte proportion (~25%) than the control treatments (~55%; Figure 8). In the Maintenance Dock 2021 experiment, treatments with NH_4_ amendments had lower proportions of heterocytes than the controls, close to values that were present in the initial surface water (Figure 8). Overall, heterocyte proportions were not correlated to biovolume estimates (Pearson's *r* = −0.13, 95% confidence interval [−0.70, 0.54] for *Aphanizomenon* and *r* = −0.30 [−0.78, 0.40] for *Dolichospermum*).

In terms of biomass (inferred from chlorophyll *a*) or biovolume, phytoplankton in P amendments were not more abundant than in the control treatment (Table 3). Not only did overall biovolume not respond to P but neither did the biovolume of individual divisions (Figure 5). Only three genera (*Fragilaria*, *Aphanizomenon*, and *Dolichospermum*) ever had any P treatment with a biovolume higher than the control (and in each of those cases, this happened in just one experiment; Figure 5).

**TABLE 3:** Amendment effects on phytoplankton community abundance, community composition, heterocyte formation and cyanotoxin RNA copies.

Site	Chlorophyll *a*	Biovolume	Community dissimilarity	*Aphanizomenon* heterocytes	*Dolichospermum* heterocytes	*mcy*E RNA	*ana*C RNA^a^	*cyr*A RNA^a^	*sxt*A RNA^a^
Ek Bay (2021)	None	NO_3_ (+)	NH_4_, P, N + P	NO_3_ (−), P (+)	P (+), N + P (−)	NH_4_ (−), N + P (−)^a^	NA	NH_4_ (−), P (+)	NH_4_ (−), NO_3_ (−), N + P (−)
Maintenance Dock (2021)	NH_4_ (+), NO_3_ (+), N + P (+, serial co‐limitation)	NH_4_ (+), NO_3_ (+)	NH_4_, N + P	NH_4_ (−), N + P (−)	NH_4_ (−), NO_3_ (−), N + P (−)	NH_4_ (−), N + P (−)	NA	NA	ND
Ellsworth Bay (2022)	NH_4_ (+), NO_3_ (+), N + P (+, serial co‐limitation)	NH_4_ (+), NO_3_ (+), N + P (+, serial co‐limitation)	P, N + P	Not measured	Not measured	NO_3_ (−), P (−), N + P (−)	NO_3_ (−), N + P (−)	ND	NO_3_ (−), N + P (−)
Maintenance Dock (2022)	NH_4_ (+), NO_3_ (+), N + P (+, serial co‐limitation)	N + P (+, simultaneous co‐limitation)	NH_4_, N + P	Not measured	Not measured	NH_4_ (+), P (−), N + P (−)^a^	ND	ND	P (+)

*Note*: Direction of effects is indicated by + (positive effect) or – (negative effect).

Abbreviations: N + P, amended with NH_4_, NO_3_ and P; NA, Not enough measurements above the detection limit to estimate effects; ND, no differences from the control indicated by statistical analysis; NH_4_, amended with ammonium; NO_3_, amended with nitrate; P, amended with orthophosphate.

^a^ Used Peto‐Peto test to compare amendments to control treatment only.

### Effects of nutrient addition on potential cyanotoxin production

DNA copies from all four cyanotoxin production genes considered in this study were observed in initial surface water used to start the experiments (*ana*C, *cyr*A, *mcy*E, *sxt*A; Figure 2b–e). Also present at the start of each experiment were *mcy*E RNA gene copies, indicating surface water communities were already expressing MC production genes in Kabetogama Lake water before the experiment started (Figure 2b). RNA copies of *cyr*A genes were only observed in initial surface water samples at one site in 2021 (Maintenance Dock), and both *ana*C and *sxt*A RNA gene copies were observed at both sites in 2022 (Figure 2c–e).

The *mcy*E RNA copies standardized by water volume and *mcy*E RNA per cyanobacteria biovolume (μm^3^) were very strongly correlated (Pearson's *r* = 0.92, confidence interval 0.88, 0.94). As a result, experimental effects were very similar regardless of whether the *mcy*E gene data were expressed per biovolume or per volume. Conversely, cyanobacterial biovolume was not correlated to *mcy*E RNA gene copies per volume or per biovolume (*r* = −0.14 [−0.33, 0.07] and *r* = −0.16 [−0.36, 0.04], respectively). This result indicates that MC production potential (inferred from *mcy*E RNA gene copies) responds differently from cyanobacterial growth.

**FIGURE 9:**
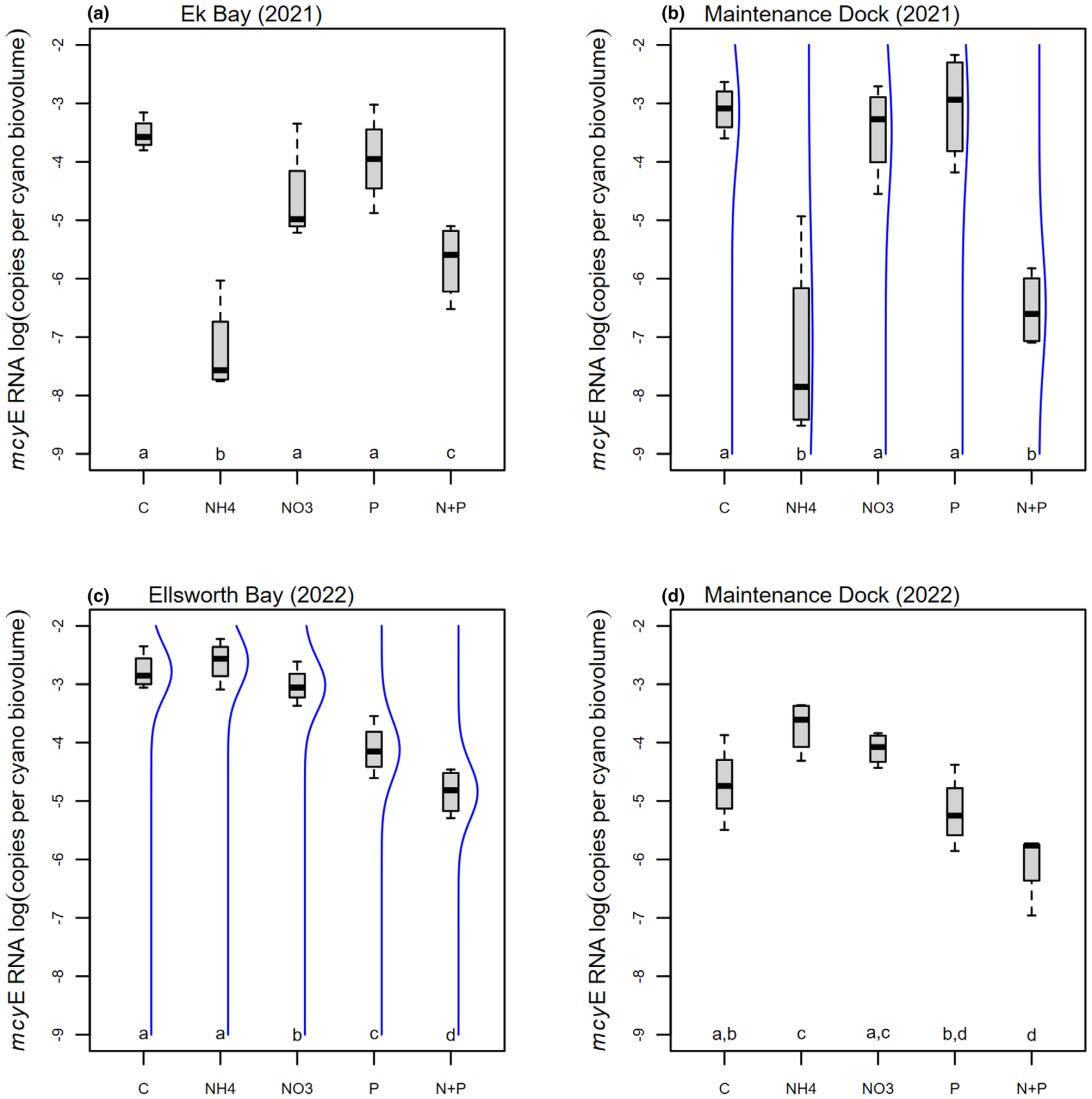
Box and whisker plot of microcystin *mcy*E RNA copies per cyanobacterial biovolume (copies per μm^3^ biovolume) in communities from Kabetogama Lake (Minnesota, United States) after experimental amendment at each site. For Eck Bay 2021 (a) and Maintenance Dock 2022 (d) experiments, a non‐parametric Peto‐Peto test was used to identify differences due to non‐normality of residual data. In (b and c), blue lines indicate the modeled distributions generated from the data. Letters indicate treatments that have overlapping distributions (i.e., differences between treatments that are not different from zero). C, Control; NH_4_, ammonium amendment; NO_3_, nitrate amendment; N + P, NH_4_ + NO_3_ + P amendment; P, orthophosphate amendment. Boxes encompass the first and third quartiles. The thick black line is the median. The lines (whiskers) show the largest or smallest observation that falls within 1.5 times the box size.

Chambers with NH_4_ amendments in both 2021 experiments had lower *mcy*E RNA gene copies per μm^3^ biovolume than in the control (both in the NH_4_‐alone amendment, and the N + P amendment; Figure 9a,b). By contrast, in 2022, NH_4_ alone either had no effect (Ellsworth Bay) or increased *mcy*E RNA gene copies per μm^3^ biovolume (Maintenance Dock; Figure 9c,d, Table 3). Ellsworth Bay *mcy*E RNA gene copies per μm^3^ biovolume declined slightly with NO_3_ amendment but had a stronger decline in treatments with P and N (Figure 8c). The Maintenance Dock site in 2022 only had declines in *mcy*E RNA gene copies per μm^3^ biovolume in the N + P treatment (Figure 9d). Normalizing the *mcy*E RNA gene copies per mL (instead of per cyanobacterial biovolume) resulted in very similar patterns (Figure S2).

Further insight into N demand and cyanotoxin production might be indicated by heterocyte formation and *mcy*E RNA gene copies. The *mcy*E RNA gene copies per mL were strongly correlated to the proportion of heterocytes in *Aphanizomenon* (Pearson's *r* = 0.81; 95% confidence interval [0.36, 0.95]) and *Dolichospermum* (*r* = 0.83; [0.42, 0.96]). These correlations were very weak if *mcy*E RNA gene copies were normalized by biovolume (*Aphanizomenon r* = 0.11 [−0.56, 0.69] and *Dolichospermum r* = 0.23 [−0.47, 0.75]), reflecting the previously mentioned weak relationship between heterocyte formation and cyanobacterial biovolume in these experiments. In the Maintenance Dock 2021 experiment, heterocyte proportion followed almost the same response pattern to nutrient amendments as *mcy*E RNA gene copies per μm^3^ biovolume, but this wasn't the case in the Ek Bay 2021 experiment (Figure 9a,b).

Patterns in other cyanotoxin‐production genes were more difficult to identify because of the presence of many values below the detection limit (Figures S3, S4 and S5). The *ana*C (anatoxin) RNA gene was almost entirely absent from samples in 2021, but in 2022, *ana*C RNA gene copies per μm^3^ biovolume were lower than controls in the NO_3_ and N + P treatment at Ellsworth Bay (Figure S3, Table 3). The *cyr*A (cylindrospermopsin) RNA gene copies per μm^3^ biovolume were higher than the controls at Ek Bay in the P treatment, and the highest concentrations of *cyr*A RNA gene copies always occurred in P treatments at all sites (Figure S4, Table 3). The *sxt*A RNA gene copies were all below the detection limit in the NH_4_ and N + P treatments at Ek Bay in 2021, despite observing at least some values above the detection limit in all the other treatments. In Ellsworth Bay (2022), *sxt*A gene copies per μm^3^ biovolume in NO_3_ and N + P were low relative to the controls (Figure S5). At the Maintenance Dock in 2022, P treatments had higher *sxt*A RNA gene copies per μm^3^ biovolume than controls (Figure S5, Table 3).

Cyanotoxin gene copies had similar responses to nutrient amendment in some experiments. In Ek Bay (2021), NH_4_‐amended chambers had reduced *mcy*E, *cyr*A, and *sxt*A gene copies per μm^3^ biovolume relative to control chambers (Table 3). In Ellsworth Bay (2022), NO_3_ and N + P amended chambers had less *mcy*E, *ana*C, and *sxt*A genes (Table 3). Treatments amended with N + P never had elevated cyanotoxin RNA copies relative to the control, and N + P treatments always had less *mcy*E gene copies than controls (Figure 9, Table 3).

## DISCUSSION

### Growth limitation in experimental chambers

Although initial TN:TP and TDN:TDP ratios were relatively high, suggesting P limitation or co‐limitation in Kabetogama Lake, accumulation of biomass or biovolume in the experimental chambers was highest in treatments with added N. The most abundant Cyanobacteria taxa were mostly genera capable of N‐fixation, and heterocytes were observed in the initial surface water that was used in the experimental setup. So why was the water column nutrient availability a poor predictor of experimental outcomes? Most of the dissolved N in the water column was not in the form of NH_4_ or NO_3_. The unaccounted‐for dissolved N could be dissolved organic N, which encompasses an array of molecules with very different lability, from urea to highly refractory humic acids (Berman & Bronk, 2003). Based on the experimental results, it appears likely that most of the dissolved N was unavailable for phytoplankton use. Experimental results more closely followed expectations when looking at NH_4_:SRP ratios (which were <12 in all experiments that indicated N limitation). Even cyanobacteria that can fix atmospheric N tend to prefer NH_4_ or NO_3_ due to the lower energetic cost, because N fixation requires cyanobacteria to invest in enzymes, specialized metabolites, management of oxygen in the cell, and sometimes specialized cells (although the specifics vary among taxa; Falkowski, 1983, Raven, 2012). This fact is consistent with our observations of *Aphanizomenon*, an N‐fixing taxa that had higher biovolumes in most treatments with added NH_4_.

The conceptual basis for thinking freshwater eutrophication is primarily a P issue is that N‐fixing cyanobacteria would presumably be able to grow beyond the limitations of the available labile N when P availability is high (Schindler, 2012; Schindler & Fee, 1974). Heterocytes increased in P‐amended treatments from Eck Bay in 2021 but not from Maintenance Dock, although short‐term experiments such as these are accurately criticized for not being a realistic representation of long‐term lake‐wide phytoplankton dynamics (Schindler, 2012).

### Nutrient effects on cyanotoxin production

Most congeners of microcystin, cylindrospermopsin, and saxitoxin are N‐rich molecules (Metcalf & Codd, 2012), and these cyanotoxins are generally considered to be secondary metabolites (Omidi et al., 2018). Principles from ecological stoichiometry would suggest that when N is limiting, cells would reduce the production of these N‐rich secondary metabolites and reprioritize N to primary metabolism and growth (Chaffin et al., 2023; Sterner & Elser, 2002). Our experiments indicated N limitation in chlorophyll *a* or biovolume in three of four experiments, yet in these communities, *mcy*E RNA gene transcripts were being produced prior to the experiment. Furthermore, in 2021 there was a strong positive correlation between investment in N acquisition (in the form of heterocyte appearance) and *mcy*E RNA gene copies. In the experiments themselves, amendments of NH_4_ had very different effects on *mcy*E gene production in 2021 and 2022. So why would cyanobacteria ever increase production of an N‐rich molecule during N limitation, and why did we see such contrasting results in 2021 and 2022? Microcystin has an ancient origin and an unclear functional role in cyanobacteria (Omidi et al., 2018; Rantala et al., 2004). Although MC has often been described as a secondary metabolite, this could be inaccurate (Wei et al., 2024). At minimum, there is strong evidence that the availability and speciation of N influences variation in MC production (Inabe et al., 2021; Krausfeldt et al., 2020) as was also the case in our study, in which investment in N fixation (as inferred from heterocyte presence) was positively correlated with investment in MC production (as inferred from *mcy*E RNA gene copies), at least in 2021. Lack of NH_4_ also means the demand for certain other elements increases as cyanobacteria build heterocytes and other metabolites to acquire NO_3_, urea, or N_2_. If MC is needed for management of those other elements (e.g., metals such as iron, nickel, and molybdenum), then that need could also be why MC is upregulated when N becomes limiting (Wei et al., 2024). Understanding the functional role of MC might improve our ability to make predictions about its production in cells.

The other reason these explanations remain confusing is that similar experiments produced different results, as we observed when comparing our experimental outcomes between 2021 and 2022 (Wei et al., 2024). In our experiments, NH_4_ amendments appeared to suppress *mcy*E gene expression in 2021 but promoted *mcy*E gene expression in 2022. The most likely explanation is probably variation in taxonomic composition between years. At the morphospecies level (i.e., the species identity that can be determined by microscopy), *Aphanizomenon* and *Dolichospermum* were major contributors to the Cyanobacterial community both years, but other morphospecies varied. For example, *Microcystis* was an important contributor (in biovolume terms) during 2021 but was absent in 2022. We did not collect any data about community composition that was more detailed than morphospecies, but it has been shown that core and secondary metabolic processes can be highly variable among strains within a single morphospecies (Yancey et al., 2023). Therefore, we assume that even within the *Aphanizomenon* we identified here, very different strain composition from 2021 to 2022 could be responsible for altering the production of MC in response to NH_4_ amendments. With regards to a large lake downstream from Kabetogama Lake, data from Natwora et al. (2025) suggested that toxicity can be driven by a relatively small subset of the overall community composition. Strain‐specific metabolic and cyanotoxin production information would improve our understanding of what leads to the production of many cyanotoxins and secondary metabolites. Alternatively, antecedent conditions that were unmeasured here could be creating cryptic variation. For example, 2021 was a very dry growing season, and 2022 was a very wet growing season in the Kabetogama Lake watershed, and it is unknown the full extent of impact this could have on the phytoplankton (e.g., changes in dissolved organic matter, light, and temperature).

### Synthesis

Enrichment bioassays such as the ones we performed here have a variety of limitations, including unrealistic stable light and temperature conditions, short periods of relatively intense agitation rather than continuous gentle mixing, and many others. The longer the experiment lasts, the less representative the experimental conditions are of the real‐world community. For this reason, we kept incubation times as short as possible while providing enough time for communities to react to the treatments. However, these are, at best, snapshots of how the real‐world community could respond. The fact that these responses were inconsistent between years is an indication that we remain far from being able to generalize our knowledge about cyanotoxin production to build predictions about novel situations. Still, the consistent story from this study and other studies is that N availability and form do appear to cause cyanobacteria to alter their MC production. Why and how that response varies across species and strains remains difficult to predict.

Evidence continues to accumulate on environmental controls over cyanobacterial growth and toxicity in lakes. Substantial evidence from a wide variety of lake ecosystems supports the interpretation that N and P availability drive variation in cyanobacterial bloom frequency and intensity (Heisler et al., 2008). However, predicting the causative drivers of cyanotoxin production remains very challenging. Microcystin is the most intensely studied cyanotoxin, and we can conclude, (1) availability and speciation of N are capable of altering MC production, (2) variation in MC production occurs among strains within the same morphospecies, (3) the environmental conditions that cause MCs to be produced also vary by strain, and (4) there continues to be interest in being able to build predictive models for MC occurrence and concentration. At present, the models that do the best job of predicting MC concentration continue to be statistical models that are either focused on specific systems or are identifying large‐scale drivers of cyanobacterial abundance and infer MC will increase with overall cyanobacterial biomass (Francy et al., 2016; United States Environmental Protection Agency, 2025). Conceptually, the relevance of statistical models is limited to the conditions in which they are calibrated, so statistical models targeting high‐impact locations require long datasets, continuous updating, and at least some local knowledge about the potentially important factors driving production.

Although HABs are generally thought of as a pelagic problem, phytoplankton in shallow nearshore areas are continually interacting with the benthos. Flux of nutrients from the sediment can sustain phytoplankton, and benthic algae can be a source of propagules to the water column. Short‐term bioassay chambers isolate the phytoplankton from the sediments, but the sediments could be critical in determining how phytoplankton behave in field conditions. In an earlier study, we showed that Kabetogama Lake sediments are a source of NH_4_, and this NH_4_ could be enough to keep N‐fixing cyanobacteria from having a major competitive advantage (Larson, Bailey, Maki, et al., 2025; Larson, Bailey, & Stelzer, 2025). Sediment influence could be less in deeper, stratified waters, but most human interaction with large lakes occurs in nearshore areas. A full understanding of the nearshore phytoplankton dynamics would probably require more explicit incorporation of sediment‐water column interactions.

## AUTHOR CONTRIBUTIONS

**James H. Larson:** Conceptualization (equal); data curation (equal); formal analysis (equal); funding acquisition (equal); investigation (equal); methodology (equal); project administration (equal); resources (equal); software (equal); supervision (equal); validation (equal); visualization (equal); writing – original draft (equal); writing – review and editing (equal). **Ryan P. Maki:** Conceptualization (equal); formal analysis (equal); funding acquisition (equal); investigation (equal); methodology (equal); project administration (equal); resources (equal); supervision (equal); writing – review and editing (equal). **Sean W. Bailey:** Data curation (equal); formal analysis (equal); investigation (equal); methodology (equal); supervision (equal); writing – review and editing (equal). **Victoria G. Christensen:** Conceptualization (equal); funding acquisition (equal); investigation (equal); methodology (equal); resources (equal); writing – review and editing (equal). **Keith A. Loftin:** Methodology (equal); resources (equal); validation (equal); writing – review and editing (equal). **Erin A. Stelzer:** Conceptualization (equal); formal analysis (equal); investigation (equal); methodology (equal); resources (equal); validation (equal); writing – review and editing (equal). **James C. Smith:** Data curation (equal); methodology (equal); resources (equal); supervision (equal); writing – review and editing (equal). **Seth A. McWhorter:** Conceptualization (equal); funding acquisition (equal); investigation (equal); methodology (equal); writing – review and editing (equal).

## Supporting information


**Figure S1.** Example images collected from an imaging flow cytometer (FlowCAM). *Dolichospermum* and *Aphanizomenon* are among the taxa that are very easy to observe in FlowCAM images. Red arrows indicate the location of heterocytes in the *Dolichospermum* or *Aphanizomenon* colonies.

**Figure S2.** Box and whisker plot of microcystin mcyE RNA copies per mL in communities from Kabetogama Lake (MN, USA) after experimental amendment at each site. Blue lines indicate the modeled distributions generated from the data. Letters indicate treatments that have overlapping distributions (i.e., differences between treatments that are not different from zero). C, Control; NH4, ammonium amendment; NO_3_, nitrate amendment; P, orthophosphate amendment; N + P – NH_4_ + NO_3_ + P amendment. Boxes encompass the first and third quartiles. The thick black line is the median. The lines (whiskers) show the largest or smallest observation that falls within 1.5 times the box size.

**Figure S3.** Copies per cyanobacterial biovolume (copies per μm^3^ biovolume) of RNA transcripts for the anatoxin‐a *ana*C gene in communities from Kabetogama Lake (Minnesota, United States) after experimental amendment at each site. Open circles are values below the detection limit and filled circles are values above the detection limit. Asterisk (*) indicates that a non‐parametric Peto‐Peto test had a *p‐*value < 0.05 when compared to the control treatment. Ek Bay had no samples above the detection limit and is therefore not shown here. C, Control; NH_4_, ammonium amendment; NO_3_, nitrate amendment; P, orthophosphate amendment; N + P, NH_4_ + NO_3_ + P amendment.

**Figure S4.** Copies per cyanobacterial biovolume (copies per μm^3^ biovolume) of RNA transcripts for the cylindrospermopsin *cyr*A gene in communities from Kabetogama Lake (Minnesota, United States) after experimental amendment at each site. Open circles are values below the detection limit and filled circles are values above the detection limit. Asterisk (*) indicates that a non‐parametric Peto‐Peto test had a *p‐*value < 0.05 when compared to the control treatment. C, Control; NH_4_, ammonium amendment; NO_3_, nitrate amendment; P, orthophosphate amendment; N + P, NH_4_ + NO_3_ + P amendment.

**Figure S5.** Copies per cyanobacterial biovolume (copies per μm^3^ biovolume) of RNA transcripts for the saxitoxin *sxt*A gene in communities from Kabetogama Lake (Minnesota, United States) after experimental amendment at each site. Open circles are values below the detection limit and filled circles are values above the detection limit. Asterisk (*) indicates that a non‐parametric Peto‐Peto test had a *p‐*value < 0.05 when compared to the control treatment. C, Control; NH_4_, ammonium amendment; NO_3_, nitrate amendment; P, orthophosphate amendment; N + P, NH_4_ + NO_3_ + P amendment.

**Table S1.** Primers and probe sequences and run conditions for cyanobacteria assays.°C, degrees Celsius; s, seconds.

**Table S2.** Standard curve characteristics for cyanobacteria assays. Dynamic range and limit of detection are reported in copies per reaction.
