# Supplementary figures and images for: Phytoplankton growth and potential cyanotoxin production differ in response to nitrogen and phosphorus amendments in late summer communities from Kabetogama Lake (Minnesota, United States)
Source: J Phycol. 2026 May 2;62(3):883–903. doi: 10.1111/jpy.70166 (PMC13280782; doi:10.1111/jpy.70166)

*Dolichospermum*

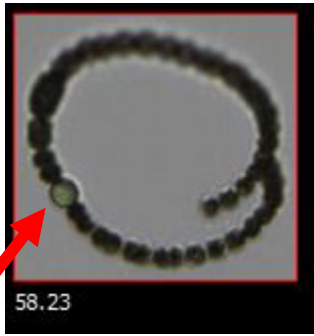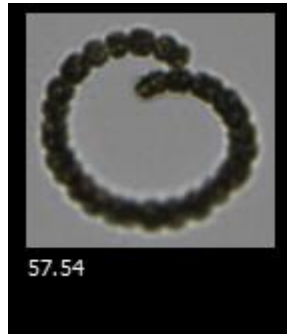

Heterocyte

Heterocytes

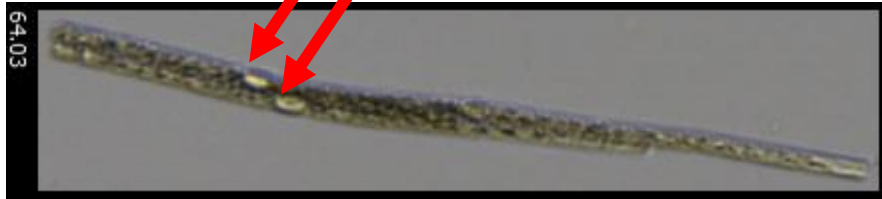

Heterocyte

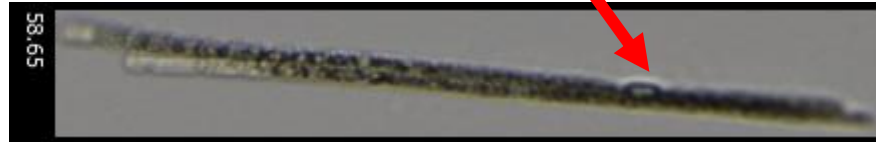

*Aphanizomenon*

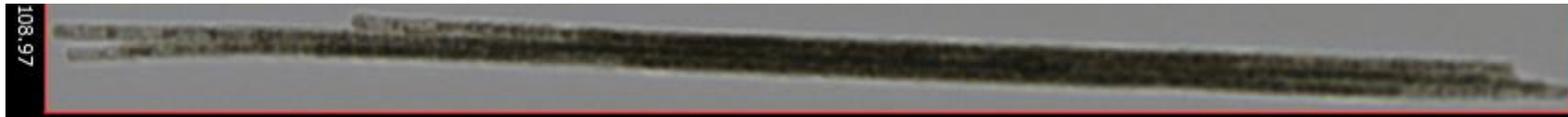

Supplement: Supplementary file 1 — Figure S1. Example images collected from an imaging flow cytometer (FlowCAM). Dolichospermum and Aphanizomenon are among the taxa that are very easy to observe in FlowCAM images. Red arrows indicate the location of heterocytes in the Dolichospermum or Aphanizomenon colonies. [file JPY-62-883-s006.pdf]

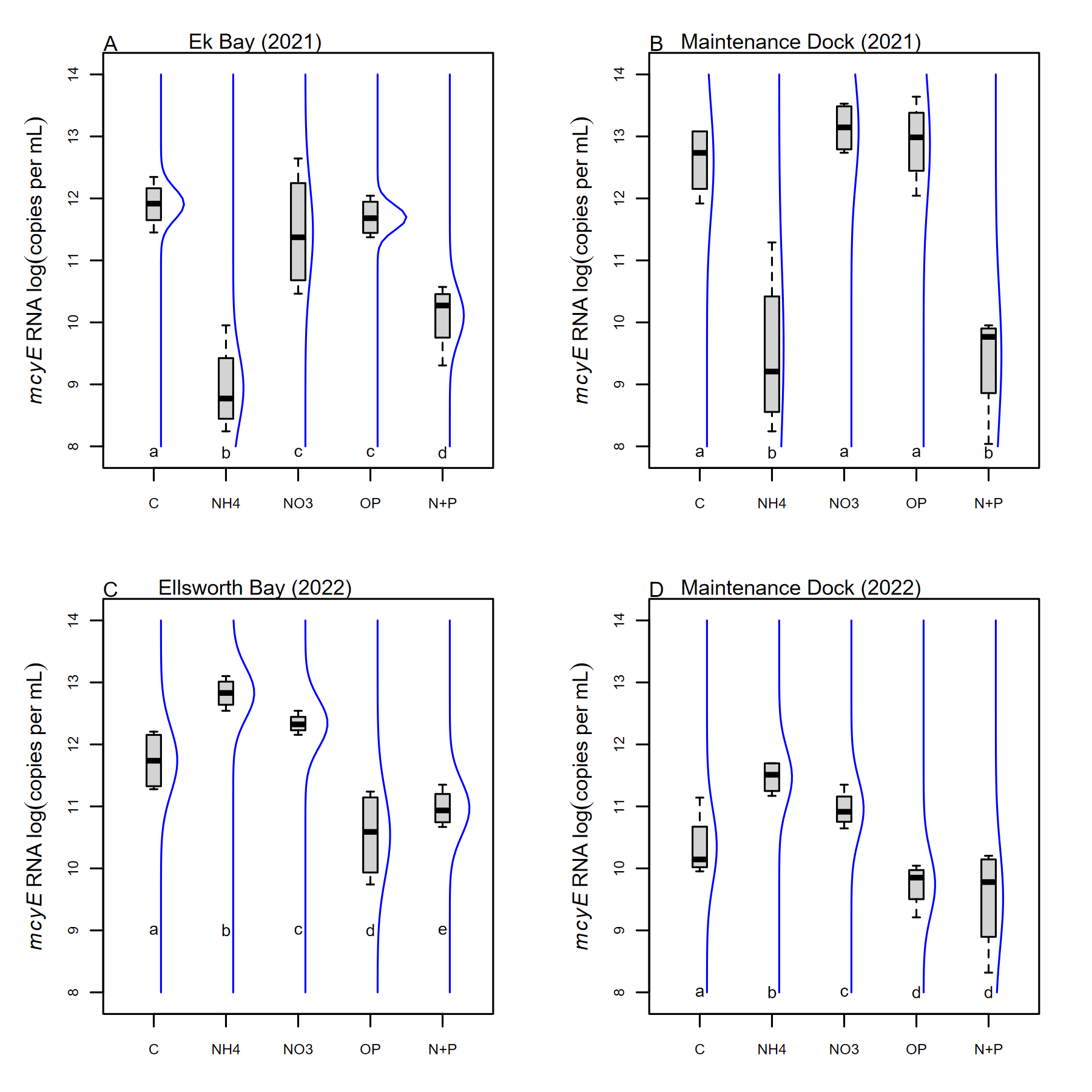

Supplement: Supplementary file 2 — Figure S2. Box and whisker plot of microcystin mcyE RNA copies per mL in communities from Kabetogama Lake (MN, USA) after experimental amendment at each site. Blue lines indicate the modeled distributions generated from the data. Letters indicate treatments that have overlapping distributions (i.e., differences between treatments that are not different from zero). C, Control; NH4, ammonium amendment; NO3, nitrate amendment; P, orthophosphate amendment; N + P – NH4 + NO3 + P amendment. Boxes encompass the first and third quartiles. The thick black line is the median. The lines (whiskers) show the largest or smallest observation that falls within 1.5 times the box size. [file JPY-62-883-s001.tiff]

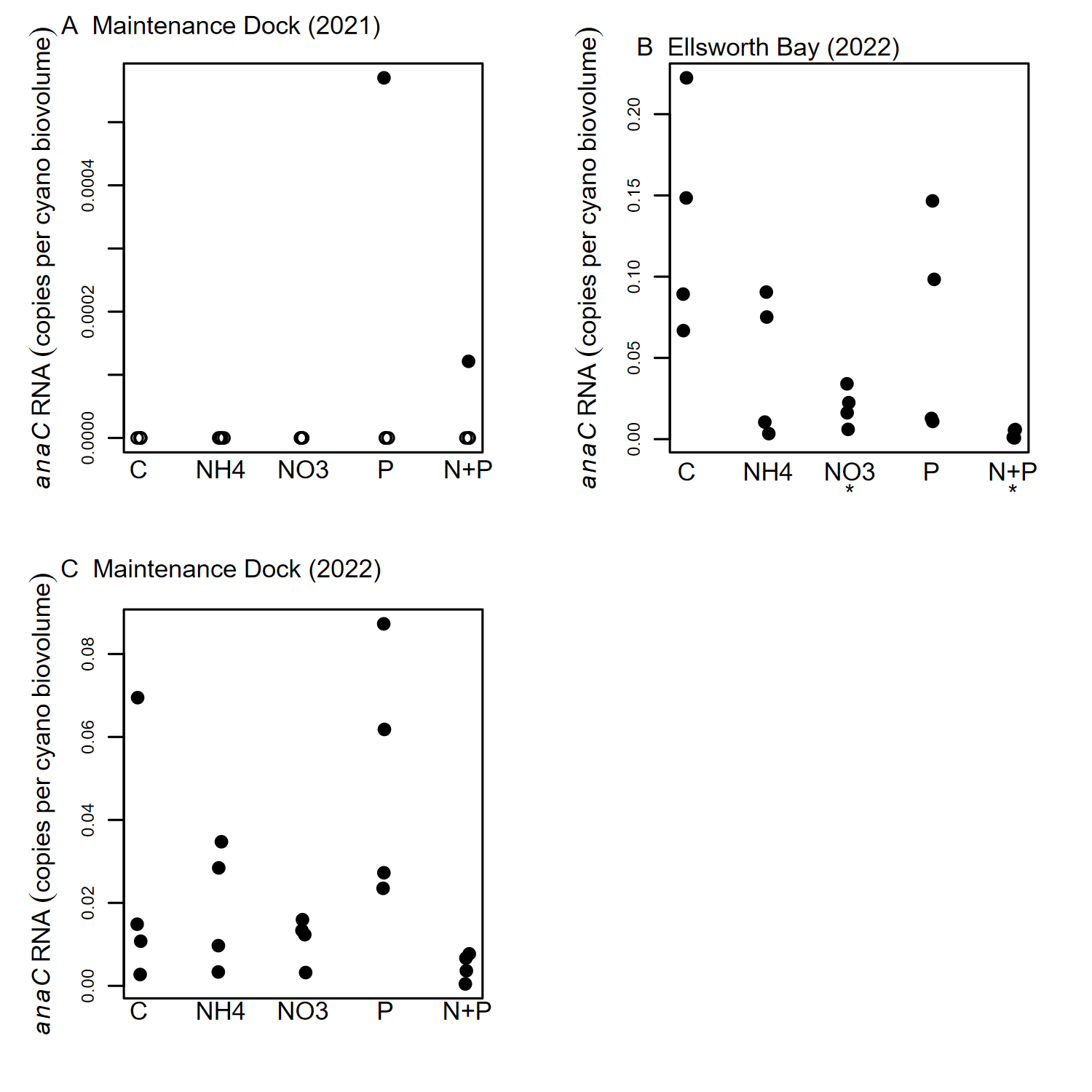

Supplement: Supplementary file 3 — Figure S3. Copies per cyanobacterial biovolume (copies per μm3 biovolume) of RNA transcripts for the anatoxin‐a anaC gene in communities from Kabetogama Lake (Minnesota, United States) after experimental amendment at each site. Open circles are values below the detection limit and filled circles are values above the detection limit. Asterisk (*) indicates that a non‐parametric Peto‐Peto test had a p‐value < 0.05 when compared to the control treatment. Ek Bay had no samples above the detection limit and is therefore not shown here. C, Control; NH4, ammonium amendment; NO3, nitrate amendment; P, orthophosphate amendment; N + P, NH4 + NO3 + P amendment. [file JPY-62-883-s007.tiff]

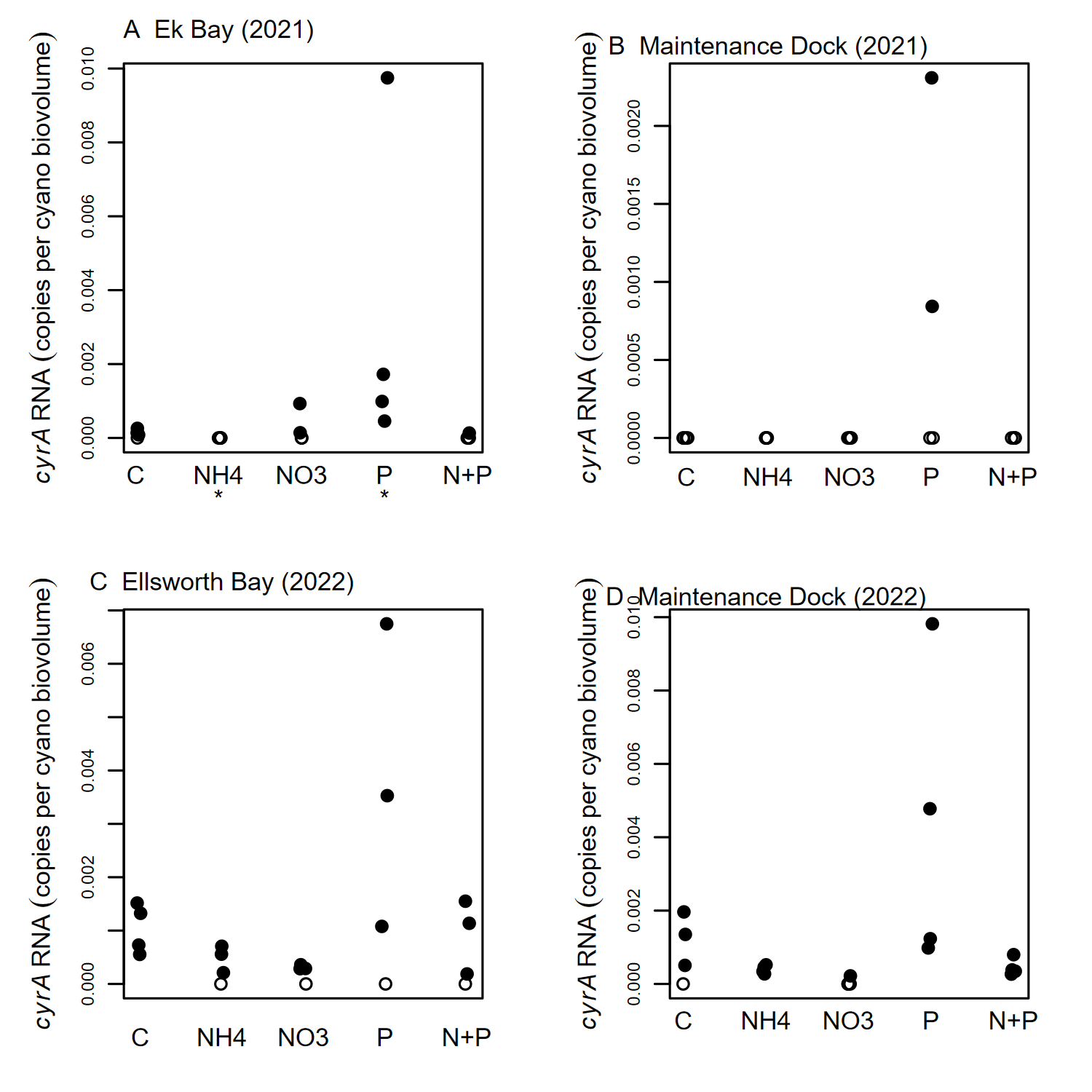

Supplement: Supplementary file 4 — Figure S4. Copies per cyanobacterial biovolume (copies per μm3 biovolume) of RNA transcripts for the cylindrospermopsin cyrA gene in communities from Kabetogama Lake (Minnesota, United States) after experimental amendment at each site. Open circles are values below the detection limit and filled circles are values above the detection limit. Asterisk (*) indicates that a non‐parametric Peto‐Peto test had a p‐value < 0.05 when compared to the control treatment. C, Control; NH4, ammonium amendment; NO3, nitrate amendment; P, orthophosphate amendment; N + P, NH4 + NO3 + P amendment. [file JPY-62-883-s004.tiff]

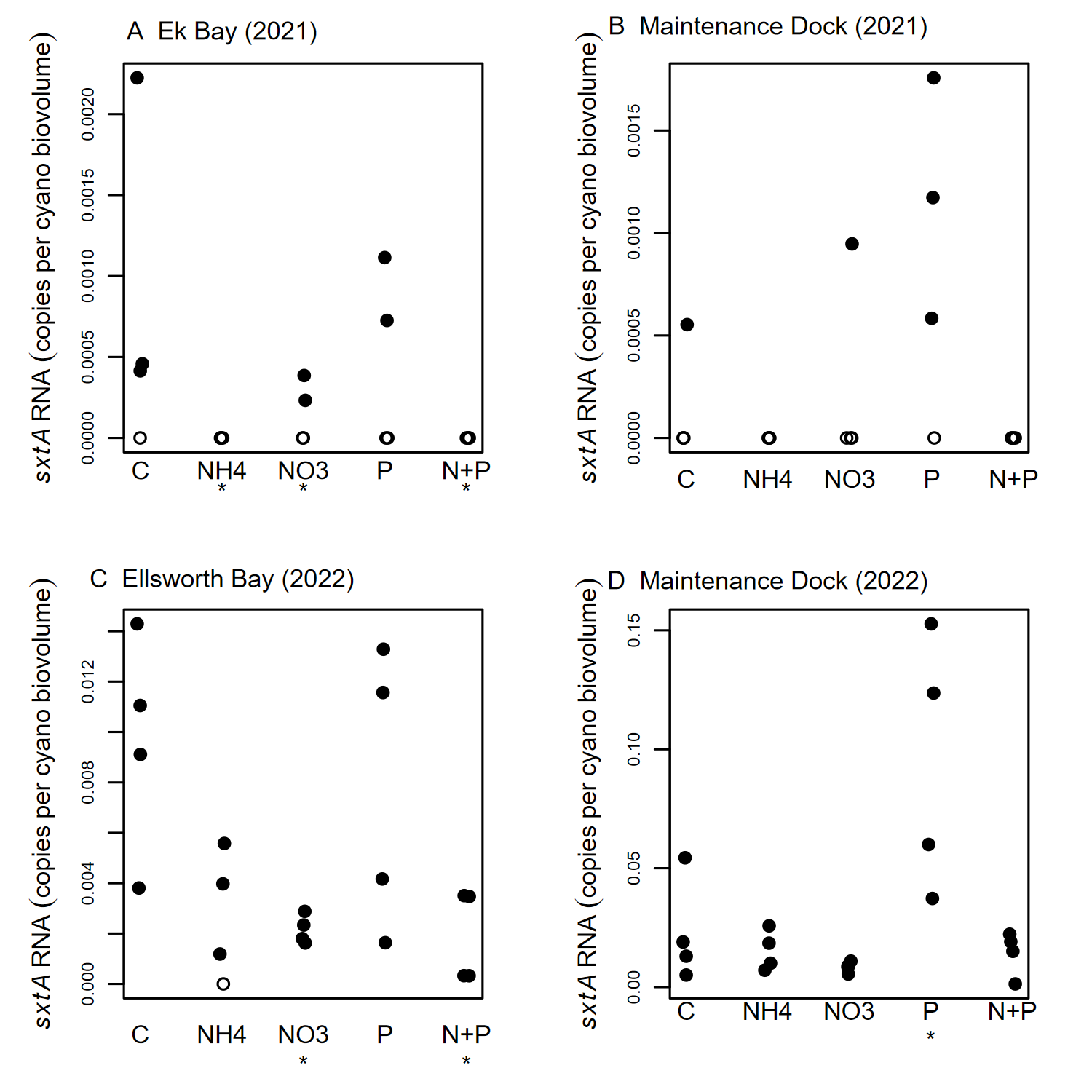

Supplement: Supplementary file 5 — Figure S5. Copies per cyanobacterial biovolume (copies per μm3 biovolume) of RNA transcripts for the saxitoxin sxtA gene in communities from Kabetogama Lake (Minnesota, United States) after experimental amendment at each site. Open circles are values below the detection limit and filled circles are values above the detection limit. Asterisk (*) indicates that a non‐parametric Peto‐Peto test had a p‐value < 0.05 when compared to the control treatment. C, Control; NH4, ammonium amendment; NO3, nitrate amendment; P, orthophosphate amendment; N + P, NH4 + NO3 + P amendment. [file JPY-62-883-s005.tiff]
