# Supplementary material for: Phytoplankton growth and potential cyanotoxin production differ in response to nitrogen and phosphorus amendments in late summer communities from Kabetogama Lake (Minnesota, United States)
Source: J Phycol. 2026 May 2;62(3):883–903. doi: 10.1111/jpy.70166 (PMC13280782; doi:10.1111/jpy.70166)
Supplement: Supplementary file 6 — Table S1. Primers and probe sequences and run conditions for cyanobacteria assays.°C, degrees Celsius; s, seconds. [file JPY-62-883-s003.docx]

| **Supplementary Table S1.** Primers and probe sequences and run conditions for cyanobacteria assays. °C, degrees Celsius; s, seconds | | |  |  |  |
| --- | --- | --- | --- | --- | --- |
|  |  |  |  |  |  |
| **Assay** | **Primer or probe** | **Sequence (5' - 3')** | **Concentration used (nanomoles)** | **Assay run conditions^a^** | **Reference** |
| anatoxin-a synthetase (*anaC*) gene | Forward primer | TCTGGTATTCAGTMCCCTCYAT |  | (94°C for 60s, 56°C for 60s, 72°C for 60s) x 45 cycles | Sabart et al., 2015 |
|  | Reverse primer | CCCAATARCCTGTCATCAA |  |  |  |
| cylindrospermopsin synthetase (*cyrA*) gene | Forward primer | GTCTGCCCACGTGATGTTATGAT |  | (95°C for 15s, 60°C for 30s) x 45 cycles | Al-Tebrineh et al., 2012 |
|  | Reverse primer | CGTGACCGCCGTGACA |  |  |  |
|  | Probe | CCTTTGGGAACGAAATTCTCGAAGCAACT |  |  |  |
| microcystin synthetase (*mcyE*) gene | Forward primer | AATAAATCATAATTTAGAACSGGVGATTTAGG |  | (95°C for 15s, 55°C for 30s, 72°C for 20s) x 45 cycles | Al-Tebrineh et al., 2012 |
|  | Reverse primer | AATAAATCATAACGRBTVADTTGRTATTCAATTTCT |  |  |  |
| saxitoxin synthetase (*sxtA*) gene | Forward primer | GGAGTGGATTTCAACACCAGAA |  | (95°C for 15s, 60°C for 30s) x 45 cycles | Al-Tebrineh et al., 2012 |
|  | Reverse primer | GTTTCCCAGACTCGTTTCAGG |  |  |  |
|  | Probe | TGCCGATTTAGAAGAAAGTATCTCTCAG |  |  |  |
| ^a^ A required hotstart activation of 95°C for 10 minutes was applied before the listed run conditions for each assay. | | |  |  |  |

Al-Tebrineh, J., Pearson, L. A., Yasar, S. A., & Neilan, B. A. (2012). A multiplex qPCR targeting hepato- and neurotoxigenic cyanobacteria of global significance. *Harmful Algae*, *15*, 19–25. <https://doi.org/10.1016/j.hal.2011.11.001>

Sabart, M., Crenn, K., Perrière, F., Abila, A., Leremboure, M., Colombet, J., Jousse, C., & Latour, D. (2015). Co-occurrence of microcystin and anatoxin-a in the freshwater lake Aydat (France): Analytical and molecular approaches during a three-year survey. *Harmful Algae*, *48*, 12–20. <https://doi.org/10.1016/j.hal.2015.06.007>
