# Supplementary material for: Phytoplankton growth and potential cyanotoxin production differ in response to nitrogen and phosphorus amendments in late summer communities from Kabetogama Lake (Minnesota, United States)
Source: J Phycol. 2026 May 2;62(3):883–903. doi: 10.1111/jpy.70166 (PMC13280782; doi:10.1111/jpy.70166)
Supplement: Supplementary file 7 — Table S2. Standard curve characteristics for cyanobacteria assays. Dynamic range and limit of detection are reported in copies per reaction. [file JPY-62-883-s002.docx]

| **Table S2.** Standard curve charateristics for cyanobacteria assays. | | |  |  |
| --- | --- | --- | --- | --- |
| Dynamic range and limit of detection are reported in copies per reaction | | |  |  |
| **Assay** | **Dynamic range** | **Average amplification efficiency (percent)** | **Average *R*^2^ value** | **Limit of detection** |
| Anatoxin-a synthetase (*anaC*) gene | 53.6–8.08E+07 | 87 | 0.998 | 3 |
| Cylindrospermopsin synthetase (*cyrA*) gene | 34.6–5.30E+07 | 97 | 0.998 | 3 |
| Microcystin synthetase (*mcyE*) gene | 22.5–3.37E+07 | 83 | 0.997 | 10 |
| Saxitoxin synthetase (*sxtA*) gene | 16.7–2.83E+07 | 89 | 0.998 | 15 |
